# Enantioselective synthesis of polyhydroxyindolizidinone and quinolizidinone derivatives from a common precursor

**DOI:** 10.3762/bjoc.10.327

**Published:** 2014-12-22

**Authors:** Nemai Saha, Shital K Chattopadhyay

**Affiliations:** 1 Department of Chemistry, University of Kalyani, Kalyani - 741235, West Bengal, India, Fax: +91+33+25828282

**Keywords:** chiral pool, dihydroxylation, indolizidines, quinolizidine, ring-closing metathesis

## Abstract

A concise asymmetric synthetic route to two new tetrahydroxyindolizidinone and quinolizidinone derivatives has been developed from a common intermediate which featured a highly selective dihydroxylation reaction and a RCM reaction as key steps.

## Introduction

**Figure 1:**
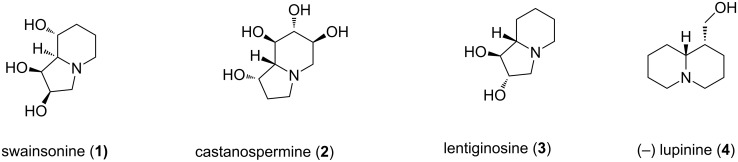
Selected polyhydroxyindolizidine and quinolizidines of importance.

Polyhydroxylated indolizidine derivatives have attracted continued interest from both organic and medicinal chemists owing to their powerful biological activities [1–3]. For example, swainsonine (**1**, Figure 1) [4] and castanospermine (**2**) [5] obtained from natural sources have potent inhibitory effects towards various glycosidase enzymes and also exhibit anti-HIV, antimetastatic, immunoregulating, antitumor, and anticancer activities [6–9]. Although naturally occurring polyhydroxylated quinolizidines are less documented, several synthetic derivatives have been prepared in the quest for analogues of the more abundant indolizidines [10–15]. Ring-size variation and/or stereochemical manipulation of the hydroxy groups have been adequately practiced for such purpose [16–17]. Indolizidines and quinolizidines with fewer hydroxy groups such as lentiginosine (**3**) [18–19] and lupinine (**4**) [20] also display a useful level of biological activities. For this, and other reasons, several novel methodologies have been developed towards the synthesis of polyhydroxylated indolizidine and quinolizidine derivatives as analogues of natural products which involved RCM [21–24], dipolar cycloaddition [25–26], nucleophilic substitution [27–28], diazo insertion [29], ring expansion–transannular cyclization [30], Cope–House cyclization [31], etc. as key steps. Although great advances have been made, creation of diverse entities from a single source remains important. Herein, we report a synthetic entry to some polyhydroxylated indolizidine and quinolizidine derivatives from a common source and involving a common set of reactions.

## Results and Discussion

**Figure 2:**
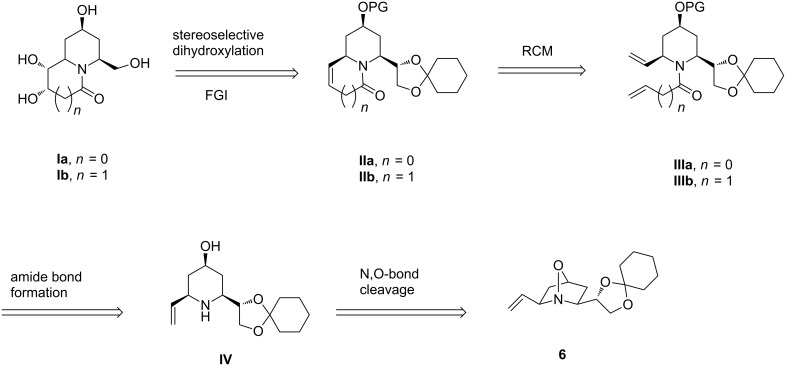
Target bicyclic imino sugars **Ia** and **Ib** from a common intermediate **IV**.

The bicyclic oxaza derivative **6** (Figure 2), previously prepared [32–33] by us from imine **5**, was identified as a starting material where the built-in functionalities at the 2,6-positions were considered suitable for the stated purpose as demonstrated retro-synthetically in Figure 2. Thus, the *cis-*hydroxy groups in the tetrahydroxyindolizidine/quinolizidine derivative represented by the general structure **I** were thought to be obtainable by a substrate-controlled hydroxylation of the corresponding cycloalkene **II** wherein the protected 1,2-dihydroxyethyl side chain would serve as precursor of the hydroxymethyl unit in **I** on functional group manipulation. The bicyclic framework of the cycloalkene **II** was expected to be obtained from a successful RCM reaction of the *N*-tethered diene **III** which, in turn, could be prepared from amide bond formation between the amine **IV** and acrylic acid (for **IIIa**) or butenoic acid (for **IIIb**). The remaining hydroxy group at C-4 could possibly be generated by a reductive cleavage of the N–O bond in **6**.

**Scheme 1:**
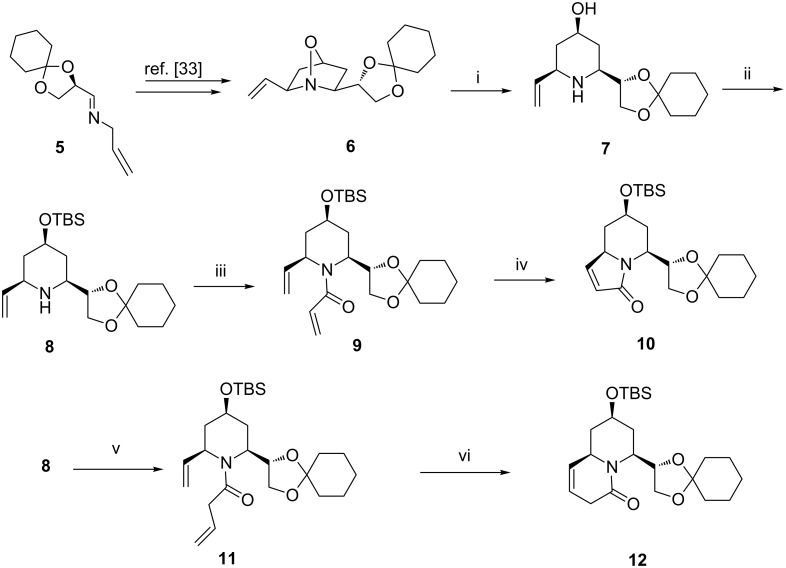
Reagents and conditions: (i) Zn/AcOH, rt, 1 h, 86%. (ii) TBSOTf, DIPEA, CH_2_Cl_2_, −5 °C, 1 h, 91%. (iii) Acrylic acid, EDC, HOBt, NMM, CH_2_Cl_2_, 0 °C to rt, 6 h, 96%. (iv) G-II (8 mol %), benzene, reflux, 24 h, 75%. (v) Vinyl acetic acid, EDC, HOBt, NMM, CH_2_Cl_2_, 0 °C to rt, 10 h, 90%. (vi) G-II (3 mol %), benzene, 50 °C, 2 h, 95%.

Thus, treatment of compound **6** with Zn/AcOH proceeded well to give the all*-cis-*piperidine derivative **7** (Scheme 1) in very good yield. The 4-OH group in compound **7** was then protected as its TBDMS ether (**8**) wherein the use of TBS triflate was essential as the more conventional TBSCl was found to be ineffective. Treatment of the free amino group in **8** with neat acrylic acid provided the unsaturated amide **9** in readiness for a subsequent RCM reaction. Ring closure of **9** proceeded better in the presence of Grubbs’ second generation catalyst [34] to provide the indolizidine derivative **10** in good yield. Similarly, treatment of amine **8** with vinylacetic acid in the presence of EDC/HOBt under standard conditions proceeded smoothly to provide the *N*-tethered diene **11**. Ring-closure of compound **11** proved to be more facile, as expected, and the quinolizidine derivative **12** was obtained in higher yield. The four step sequences **6**→**10** and **6**→**12** proceeded in overall yields of 56% and 67%, respectively.

**Scheme 2:**
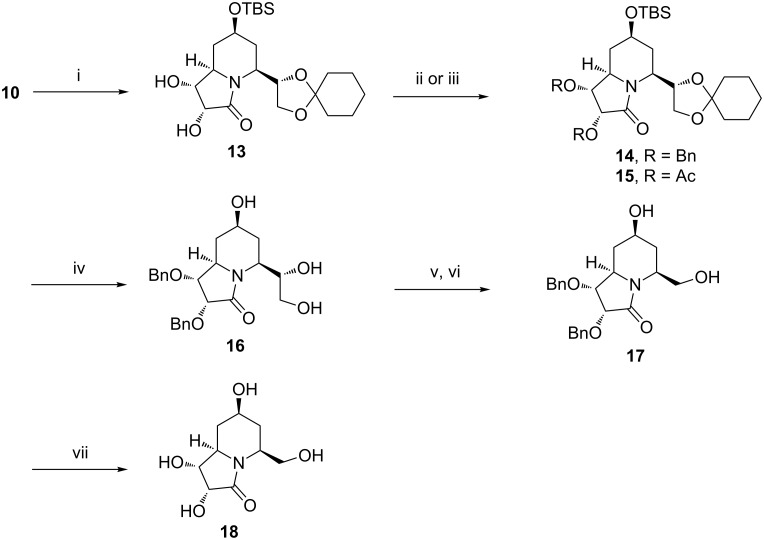
Reagents and conditions: (i) OsO_4_, NMO, acetone/water, rt, 12 h, 96%. (ii) NaH, THF, BnBr, Bu_4_NI, 0 °C to rt, 6 h, 70%. (iii) Ac_2_O, pyridine, rt, 12 h, 80%. (iv) HCl (2 N), THF, 18 h, 89%. (v) NaIO_4_, CH_3_CN/H_2_O, 5–10 °C, 30 min, (vi) NaBH_4_, MeOH, 0°C to rt, 30 min, 92% over two steps. (vii) H_2_, Pd(OH)_2_/C, MeOH, 3 h, 81%.

Having secured quick access to the unsaturated indolizidinone and quinolizidinone ring systems **10** and **12**, we considered their conversion to the desired polyhydroxylated targets through dihydroxylation of the double bond. Pleasingly, dihydroxylation of compound **10** proceeded well under Upjohn conditions [35] and provided a single isomer **13** (Scheme 2) in high yield (96%). The high selectivity in the dihydroxylation step is noteworthy as in similar situations mixture of diastereomers has occasionally been formed [36–37].

**Figure 3:**
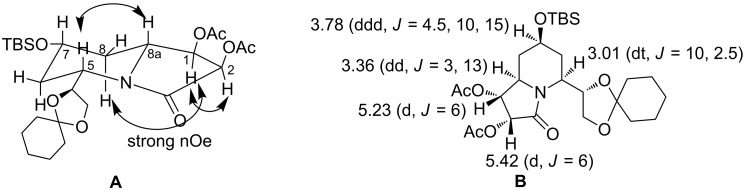
Selected nOe correlations (A) and coupling constants (B) for compound **15**.

The stereochemical identity of the newly formed stereogenic centres in **13** could not be ascertained at this stage due to the lack of well-resolved NMR data. To this end, the corresponding *O*-benzylated derivative **14** and the *O*-acetyl derivative **15** were prepared. Disappointingly, compound **14** proved to be of no advantage in this regard. On the contrary, the diacetyl derivative **15** revealed interesting ^1^H NMR and NOESY data which are summarized in Figure 3 (A and B).

**Figure 4:**
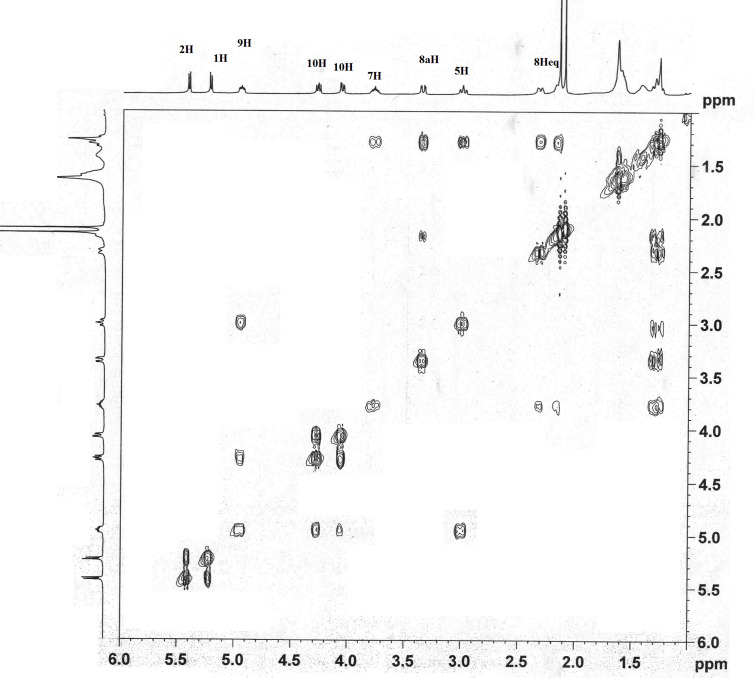
^1^H,^1^H COSY spectrum of compound **15**.

A strong nOe between the protons 8-H and 1-H (**A**), 5-H and 8a-H, 1-H and 2-H as well as the absence of a nOe between 8a-H and 1-H led us to conclude that dihydroxylation has taken place from the α-face as expected. The ^1^H,^1^H COSY experiment (Figure 4) further revealed that the absence of the correlation between the protons 1-H and 8a-H indicating a bisecting dihedral angle and hence a coupling between these two protons in the ^1^H NMR was not observed. These data clearly established the stereochemistry of **15** as depicted.

The *O*-benzylated compound **14**, however, proved to be more useful in the subsequent synthetic sequence. Thus, HCl-mediated deprotection of the acetal unit in **14** resulted in simultaneous removal of the silyl protecting group leading to the triol derivative **16** in an impressive yield of 89%. One-pot oxidative cleavage of the vicinal diol unit in the latter to a formyl group (not isolated) followed by its in situ reduction with NaBH_4_ delivered the hydroxymethyl chain in **17**. Hydrogenolytic removal of the two benzyl ether functionalities with Pearlman’s catalyst then afforded the tetrahydroxyindolizidine derivative **18**.

**Scheme 3:**
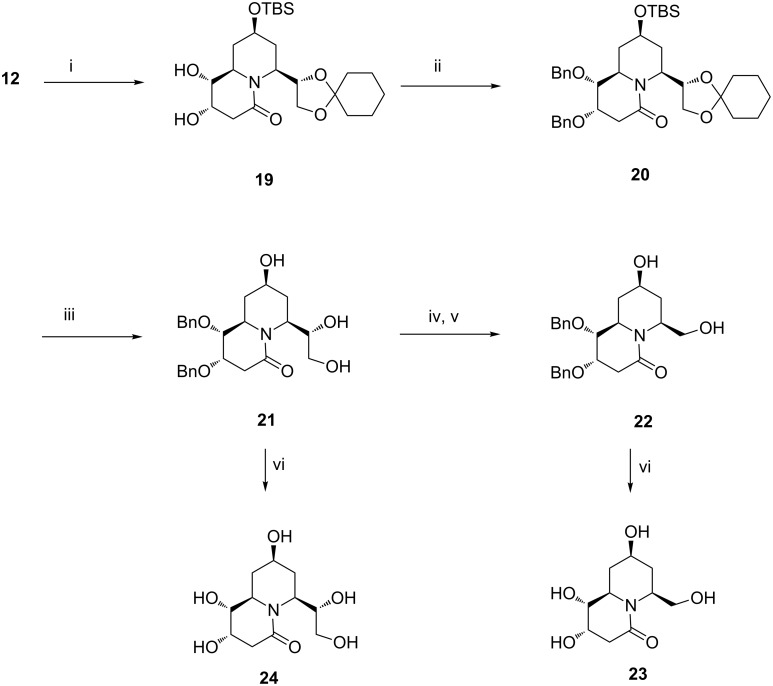
Reagents and conditions: (i) OsO_4_, NMO, acetone/water, 6 h, 95%. (ii) NaH, THF, BnBr, Bu_4_NI, 0 °C to rt, 6 h, 82%. (iii) HCl (2 N), THF, 12 h, 80%. (iv) NaIO_4_, CH_3_CN/H_2_O, 5–10 °C, 30 min; (v) NaBH_4_, MeOH, 0 °C to rt, 30 min, 90% over two steps. (vi) H_2_/Pd(OH)_2_-C, MeOH, 6 h, 80% for **23** and 85% for **24**.

Similarly, in an effort towards the preparation of tetrahydroxyquinolizidine derivatives, we considered dihydroxylation of the unsaturated quinolizidine derivative **12**. Pleasingly, dihydroxylation of **12** proved to be more facile and rewarding as it also led to the formation of a single isomer **19** (95% yield, Scheme 3). Repetition of the synthetic sequence on **19** detailed for the conversion **13**→**18**, i.e., protection of the diol as its dibenzylic ether **20**, acid-mediated one-pot deprotection of the acetal and silyl moieties leading to the triol **21**, redox manipulation of the vicinal diol unit in the latter to a hydroxymethyl unit, and subsequent debenzylation of the resulting **22** led to the desired tetrahydroxyquinolizidine derivative **23** in an overall yield of 45% over six steps. Similarly, the pentahydroxylated quinolizidine derivative **24** was prepared from the triol **21** in view of the importance of such compounds having a dihydroxyethyl side chain.

**Figure 5:**
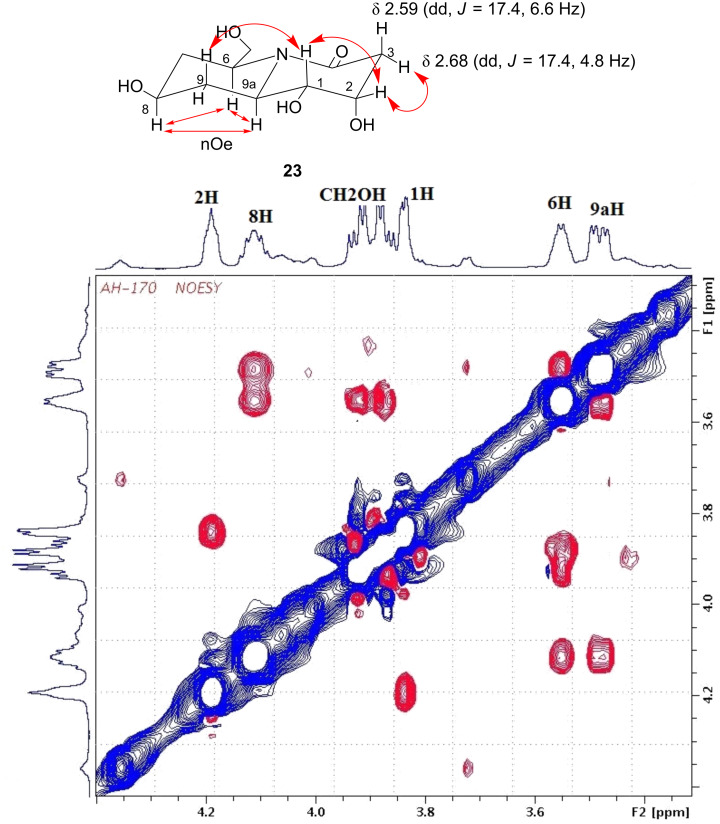
Selected nOe correlations and part NOESY spectrum of compound **23** in D_2_O (600 MHz).

The stereochemistry of the dihydroxylation reaction could not be adequately confirmed from NMR-spectroscopic measurements on **19**. However, corresponding data on **23** revealed distinct coupling patterns in 3-H (δ 2.59, dd, *J* = 17.4, 6.6 Hz; δ 2.68 dd, *J* = 17.4, 4.8 Hz) protons as well as strong nOe between the protons on 6-H and 9a-H, 6-H and 8-H, 8-H and 9a-H, 1-H and 2-H and 2-H and 3-H as indicated in Figure 5. These studies led us to believe [38] the molecular conformation of **23** to be ^5^C_8_.

## Conclusion

In conclusion, we have developed an efficient synthetic route to prepare polyhydroxylated indolizidinone and quinolizidinone derivatives of potential importance, **18**, **23** and **24** in overall yields of 25, 30 and 35%, respectively, from a single source in a linear sequence of nine steps. The methodology developed is a simple and concise one and hence may complement to those existing in the literature. The prepared compounds may also prove to be biologically important.

## Supporting Information

File 1 Experimental details and analytical data of all new compounds as well as their ^1^H and ^13^C NMR spectra.
