# Supplementary material for: Enantioselective synthesis of polyhydroxyindolizidinone and quinolizidinone derivatives from a common precursor
Source: Beilstein J Org Chem. 2014 Dec 22;10:3104–10. doi: 10.3762/bjoc.10.327 (PMC4311665; doi:10.3762/bjoc.10.327)
Supplement: File 1 — Experimental details and analytical data of all new compounds as well as their 1H and 13C NMR spectra. [file Beilstein_J_Org_Chem-10-3104-s001.pdf]

Supporting Information

for

Enantioselective synthesis of polyhydroxyindolizidinone and quinolizidinone derivatives from a common precursor

Nemai Saha and Shital K. Chattopadhyay*

Address: Department of Chemistry, University of Kalyani, Kalyani - 741235, West Bengal, India, Fax: +91+33+25828282

Email: Shital K. Chattopadhyay - skchatto@yahoo.com

*Corresponding author

Experimental details and analytical data of all new compounds as well as their ^{1}H and ^{13}C NMR spectra

Content **pages**

Experimental procedures and characterization data for all new compounds……………….….S2–S15
Copies of NMR spectral data for all new compounds ……………………………………..…S16–S38

Experimental procedures and characterization data for all new compounds

Column chromatography was performed on silica gel, Merck grade (230-400 mesh). TLC plates were visualized with vaniline solution, in an iodine chamber or with UV, unless noted otherwise. Melting points were recorded in open capillaries and are uncorrected. Optical rotations were measured on a Rudolph Autopol-IV polarimeter purchased from a DST grant and IR spectra were recorded on a Perkin-Elmer Spectrum-1 instrument using KBr disks, chloroform solution or as neat. ^{1}H NMR and ^{13}C NMR spectra were recorded on a Bruker Avance 400 and 600, operating at 400, 600 MHz for ^{1}H and 100 and 150 MHz for ^{13}C NMR, respectively purchased from a DST-FIST grant. HRMS were performed in a JEOL-JNM mass spectrometer obtained from a paid source. Dichloromethane was distilled over calcium hydride under an inert atmosphere. THF, toluene, benzene and ether were freshly distilled under argon from a purple solution of sodium and benzophenone ketyl. Unless stated otherwise, all reagents were purchased from commercial sources and used without additional purification.

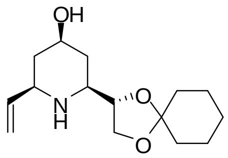

(2*S*,4*R*,6*R*)-2-((*S*)-1,4-Dioxaspiro[4.5]decan-2-yl)-6-vinylpiperidin-4-ol (7).

A solution of oxazabicycle **6** (200 mg, 0.75 mmol) in acetic acid/water (v/v 80:20, 2.0 mL) was treated with activated zinc powder (245 mg, 3.75 mmol) portion wise at room temperature for 1 h. Water (2 mL) was then added, and the mixture was filtered. The filter cake was washed with water (5 mL) and the combined aqueous solution was neutralized with solid $NaHCO_3$. The aqueous mixture was then repeatedly extracted with EtOAc (3×10 mL) and the combined organic phase was washed with water (1×10 mL), brine solution (1×10 mL), and then dried over $MgSO_4$. The dried solution was then filtered, and the filtrate was concentrated under reduced pressure to leave a crude product which was purified by column chromatography over neutral alumina using ethyl acetate-hexane mixture (1:1) to give the piperidinol **7** as a colorless liquid (177 mg, 86%). $[\alpha]_D$ +6.9 (*c* 0.85, $CHCl_3$); IR ($CHCl_3$): 3392, 2936, 2858, 1645, 1449, 1367, 1281, 1163, 1100, 1040, 928 cm^{-1}; ^{1}H NMR ($CDCl_3$, 400 MHz): δ = 5.84 (ddd, J = 6.4, 10.4, 16.8 Hz, 1 H), 5.18 (dd, J = 1.2, 16.8 Hz, 1 H), 5.05 (dd, J = 0.8, 10.0 Hz, 1 H), 4.09-4.04 (m, 1 H), 3.96 (d, J = 6.4 Hz, 2 H), 3.73 (dq, J = 4.4, 10.8 Hz, 1 H), 3.17-3.13 (m, 1 H), 2.90-2.86 (m, 1 H), 2.05-1.97 (m, 2 H), 1.77 (brm, 2 H),

1.62-1.58 (m, 8 H), 1.41-1.32 (m, 2 H), 1.17 (dd, J = 11.6, 22.8 Hz, 1 H), 1.00 (dd, J = 11.6, 22.8 Hz, 1 H). ^{13}C NMR ($CDCl_3$, 100 MHz): δ = 140.4, 114.6, 109.5, 78.0, 69.0, 64.9, 57.2, 55.4, 41.5, 36.9, 36.0, 34.7, 25.1, 24.0, 23.8; HRMS (QTOF ES+) found *m/z* 268.1906 $(M+H)^+$; $C_{15}H_{26}NO_3$ requires 268.1913.

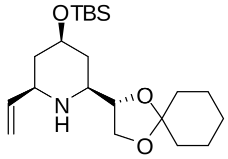

(2*S*,4*R*,6*R*)-4-(*tert*-Butyldimethylsilyloxy)-2-((*S*)-1,4-dioxaspiro[4.5]decan-2-yl)-6-vinylpiperidine (8).

DIEA (145 mg, 190 µl, 1.12 mmol) followed by TBS-OTf (295 mg, 260 µl, 1.12 mmol) was added drop wise to a stirred solution of the aminol **7** (200 mg, 0.75 mmol) in dry CH_2Cl_2 (5 mL) at −5 °C. The resulting mixture was stirred for 1 h at the same temperature before being quenched with dry methanol (1 mL). The reaction mixture was then diluted with CH_2Cl_2 (25 mL) and the combined organic solution was successively washed with water (1×25 mL), brine solution (1×25 mL) and then dried over $MgSO_4$. The dried solution was then filtered and the filtrate was concentrated in *vacuo* to leave a crude product, which was purified by flash chromatography over silica gel using 5% ethyl acetate in hexane to give the silyl ether **8** (260 mg, 91%) as a colorless oil. $[\alpha]_D$ +5.9 (*c* 0.48, $CHCl_3$). IR ($CHCl_3$): 3436, 2936, 2857, 1645, 1463, 1448, 1362, 1252, 1163, 1099, 926, 837, 775 cm^{-1}. ^{1}H NMR ($CDCl_3$, 400 MHz): δ = 5.76 (ddd, J = 6.8, 10.4, 17.2 Hz, 1 H), 5.10 (dd, J = 0.8, 17.2 Hz, 1 H), 4.95 (dd, J = 0.8, 10.8 Hz, 1 H), 4.00-3.86 (m, 3 H), 3.60 (sep, J = 4.4 Hz, 1 H), 3.06-3.02 (m, 1 H), 2.83-2.79 (m, 1 H), 1.80-1.73 (m, 2 H), 1.55-1.51 (m, 10 H), 1.35-1.31 (m, 1 H), 1.14 (dd, J = 11.6, 23.2 Hz, 1 H), 0.94 (dd, J = 11.6, 23.2 Hz, 1 H), 0.82 (s, 9 H), -0.01 (s, 6 H). ^{13}C NMR ($CDCl_3$, 100 MHz): δ = 140.9, 114.1, 109.2, 78.2, 69.8, 64.8, 57.2, 55.1, 42.2, 37.6, 36.0, 34.8, 25.8, 25.2, 23.9, 23.8, 18.1, - 4.6; HRMS (QTOF ES+) found *m/z* 382.2774 $(M+H)^+$; $C_{21}H_{40}NO_3Si$ requires 382.2777.

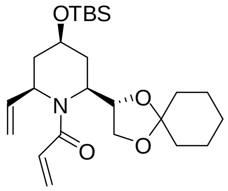

1-((2*S*,4*R*,6*R*)-4-(*tert*-Butyldimethylsilyloxy)-2-((*S*)-1,4-dioxaspiro[4.5]decan-2-yl)-6-vinylpiperidin-1-yl)prop-2-en-1-one (9).

EDC (200 mg, 1.05 mmol), acrylic acid (58 mg, 55 μL, 0.8 mmol), *N*-methylmorpholine (55 μL, 0.53 mmol) and HOBt (70 mg, 0.53 mmol) were sequentially added to a stirred solution of the amine **8** (200 mg, 0.53 mmol) in dry CH_2Cl_2 (5 mL) at 0 °C. The reaction mixture was then allowed to come to room temperature and stirred for 6 h. The reaction mixture was then extracted with CH_2Cl_2 (2×25 mL) and the combined extract was washed sequentially with 1 (N) HCl (1×20 mL), saturated aqueous $NaHCO_3$ solution (1×20 mL), water (1×20 mL) and brine (1×20 mL). The resulting solution was then dried over anhydrous $MgSO_4$, filtered, and the filtrate was concentrated in *vacuo* to leave a crude product which was purified by flash chromatography over silica gel using 1:8 ethyl acetate-hexane solution to give the amide **9** as a colourless oil (220 mg, 96%). $[\alpha]_D$ – 4.3 (*c* 0.86, $CHCl_3$); IR ($CHCl_3$): 2935, 2858, 1649, 1613, 1420, 1366, 1254, 1163, 1101, 1067, 928, 910, 837, 776 cm^{-1}; ^1H NMR ($CDCl_3$, 400 MHz): δ = 6.60-6.47 (m, 1 H), 6.24-6.07 (m, 2 H), 5.64-5.60 (m, 1 H), 5.34-4.84 (m, 3 H), 4.51-4.43 (m, 1 H), 3.97-3.62 (m, 4 H), 2.11 (br m, 1 H), 1.82-1.80 (m, 2 H), 1.65-1.45 (m, 9 H), 1.30-1.18 (m, 2 H), 0.81 (s, 9 H), -0.001 (s, 3 H), -0.02 (s, 3 H); ^1H NMR (DMSO-d_6, 400 MHz, 70 °C): δ = 6.71-6.62 (m, 1 H), 6.05 (br d, J = 16.4 Hz, 2 H), 5.63 (d, J = 10.0 Hz, 1 H), 5.00-4.97 (m, 2 H), 4.71-4.48 (m, 3 H), 4.03 (br s, 1 H), 3.71 (dd, J = 6.4, 8.0 Hz, 1 H), 3.65 (br m, 1 H), 2.0-1.92 (m, 1 H), 1.82-1.58 (m, 3 H), 1.48-1.36 (m, 8 H), 1.28-1.16 (m, 2 H), 0.80 (s, 9 H), -0.04 (s, 3 H), -0.05 (s, 3 H). ^{13}C NMR ($CDCl_3$, 100 MHz): δ = 167.5 (166.6), 140.7 (139.9), 128.9 (128.1), 116.9 (115.1), 109.8, 67.2, 65.8, 65.0, 54.3, 49.6 (48.8), 37.4, 36.4, 35.5, 34.6, 32.3, 31.3, 25.8, 25.2, 24.0, 23.9, 18.1, -4.9, -5.1. ^{13}C NMR (DMSO-d_6, 100 MHz, 70 °C): δ = 166.4, 141.1, 129.2, 127.7, 114.8, 108.8, 76.7, 66.2, 64.6, 52.6, 48.8, 38.8, 36.4, 34.7, 31.1, 25.6, 24.6 (two signals), 23.7, 17.6, -5.2. HRMS (QTOF ES+) found *m/z* 436.2875 $(M+H)^+$; $C_{24}H_{42}NO_4Si$ requires 436.2883.

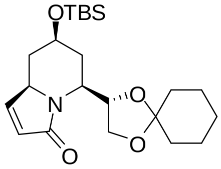

(5*S*,7*R*,8*aR*)-7-(*tert*-Butyldimethylsilyloxy)-5-((*S*)-1,4-dioxaspiro[4.5]decan-2-yl)-6,7,8,8a-tetrahydroindolizin-3(5*H*)-one (10).

Grubbs' 2nd generation catalyst (31 mg, 8 mol %) was added to a stirred solution of the diene **9** (200 mg, 0.46 mmol) in dry degassed benzene (15 ml) under argon atmosphere and the homogeneous mixture was heated to reflux for 24 h. The reaction mixture was allowed to come to room temperature and then concentrated in *vacuo* to leave a crude product which on chromatography over silica gel using ethyl acetate–petroleum ether (2:8) provided the product **10** as a colorless solid (140 mg, 75%). Mp: 118-120 °C; $[\alpha]_D$ −12.3 (*c* 0.89, $CHCl_3$); IR ($CHCl_3$): 2931, 2858, 1672, 1471, 1450, 1417, 1390, 1291, 1164, 1121, 1085, 935, 843, 775 cm^{-1}; ^1H NMR ($CDCl_3$, 400 MHz): δ = 6.99 (dd, *J* = 1.2, 5.6 Hz, 1 H), 6.0 (d, *J* = 5.6 Hz, 1 H), 5.05 (ddd, *J* = 2.8, 6.0, 9.6 Hz, 1 H), 4.34 (dd, *J* = 6.0, 9.6 Hz, 1 H), 4.01 (dd, *J* = 3.2, 9.6 Hz, 1 H), 3.90-3.85 (m, 2 H), 3.21-3.16 (m, 1 H), 2.39-2.35 (m, 1 H), 2.23-2.20 (m, 1 H), 1.64-1.61 (m, 8 H), 1.42-1.41 (m, 2 H), 1.30 (dd, *J* = 11.6, 23.2 Hz, 1 H), 1.10 (dd, *J* = 11.6, 23.2 Hz, 1 H), 0.91 (s, 9 H), 0.1 (overlapped singlet, 6 H); ^{13}C NMR ($CDCl_3$, 100 MHz): δ = 168.7, 146.2, 128.1, 109.7, 74.9, 69.6, 69.0, 61.7, 58.3, 40.2, 39.0, 37.6, 34.2, 25.8, 25.2, 24.2, 23.7, 18.1, -4.6, -4.7. HRMS (QTOF ES+) found *m/z* 430.2391 $(M+Na)^+$; $C_{22}H_{37}NNaO_4Si$ requires 430.2390.

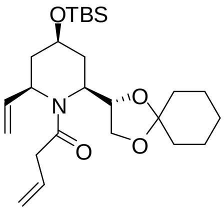

1-((2*S*,4*R*,6*R*)-4-(*tert*-Butyldimethylsilyloxy)-2-((*S*)-1,4-dioxaspiro[4.5]decan-2-yl)-6-vinylpiperidin-1-yl)but-3-en-1-one (11).

EDC (300 mg, 1.58 mmol), vinyl acetic acid (86 μL, 1.02 mmol), *N*-methylmorpholine and HOBt (106 mg, 0.79 mmol) were sequentially added to a stirred solution of the amine **8** (300 mg, 0.79 mmol) in dry CH_2Cl_2 (8 mL) at 0 °C. The reaction mixture was allowed to come to room temperature and stirred for 10 h. The resulting mixture was then extracted with CH_2Cl_2 (2×25 mL) and the combined extract was washed sequentially with 1 (N) HCl (1×20 mL),

saturated aqueous $NaHCO_3$ solution (1×20 mL), water (1×20 mL) and brine (1×20 mL). The residual solution was then dried over anhydrous $MgSO_4$, filtered, and the filtrate was concentrated in *vacuo* to leave a crude product which was purified by flash chromatography over silica gel using 1:9 ethyl acetate-hexane solution to give the amide **11** as colorless oil (318 mg, 90%). $[\alpha]_D$ –19.1 (*c* 0.83, $CHCl_3$); IR ($CHCl_3$): 2938, 2856, 1652, 1611, 1422, 1254, 1160, 1101, 910, 834, 776 cm^{-1}; ^1H NMR (mixture of rotamers) ($CDCl_3$, 400 MHz): δ = 6.14-6.08 (m, 1 H), 5.94-5.86 (m, 1 H), 5.14-4.97 (m, 4 H), 4.81-4.78 (m, 1 H), 4.56-4.52 (m, 1 H), 4.36-4.35 (m, 1 H), 4.04-3.97 (m, 1 H), 3.84-3.63 (m, 2 H), 3.20-3.03 (m, 2 H), 2.12-2.03 (m, 1 H), 1.85-1.75 (m, 2 H), 1.66-1.41 (m, 9 H), 1.31-1.30 (m, 2 H), 0.83 (s, 9 H), 0.06-(-0.06) (m, 6 H); ^1H NMR (DMSO-d_6, 400 MHz, 70 °C): δ = 6.08-6.00 (m, 1 H), 5.89-5.78 (m, 1 H), 5.19-4.96 (m, 4 H), 4.67-4.48 (m, 3 H), 4.01 (d, *J* = 3.2 Hz, 1 H), 3.70 (d, *J* = 7.2 Hz, 1 H), 3.59-3.56 (m, 1 H), 3.05-2.93 (m, 2 H), 1.94 (d, *J* = 11.6 Hz, 1 H), 1.83-1.72 (m, 3 H), 1.46-1.39 (m, 8 H), 1.27-1.18 (m, 2 H), 0.8 (s, 9 H), -0.01 (s, 3 H), -0.02 (s, 3 H).^{13}C NMR ($CDCl_3$, 100 MHz): δ = 171.3 (170.8), 140.5 (139.9), 131.9 (131.7), 117.6 (117.4), 115.1, 110.2 (109.7), 77.5 (76.6), 67.1, 65.2 (64.9), 54.3 (53.9), 49.5 (48.5), 39.2 (39.1), 37.7 (37.2), 36.3, 35.5, 35.1 (34.7), 32.0 (31.4), 25.8, 25.2 (25.16), 23.94 (23.90), 18.0, -5.0, -5.1. ^{13}C NMR (DMSO-d_6, 100 MHz, 70 °C): δ = 171.2, 141.4, 133.4, 117.3, 115.4, 109.3, 77.2, 66.7, 65.1, 53.3, 49.1, 38.5, 36.8, 32.3, 35.3, 26.1 (two signals), 25.1, 24.1, 23.9, 18.1, -4.7. HRMS (QTOF ES+) found *m/z* 472.2853 (M + Na)$^+$; $C_{25}H_{43}NNaO_4Si$ requires 472.2859.

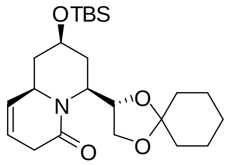

(2*R*,4*S*,9a*R*)-2-(*tert*-Butyldimethylsilyloxy)-4-((*S*)-1,4-dioxaspiro[4.5]decan-2-yl)-3,4,7,9a-tetrahydro-1H-quinolizin-6(2*H*)-one (12).

Grubbs' 2nd generation catalyst (11 mg, 3 mol%) was added to a stirred solution of the diene **11** (200 mg, 0.45 mmol) in dry degassed benzene (15 mL) under an argon atmosphere and the homogeneous mixture was heated at 50 °C for 2 h. The reaction mixture was allowed to come to room temperature and then concentrated in *vacuo* to leave a crude product which was purified by flash chromatography over silica gel using ethyl acetate–hexane (2:8) to provide the product **12** as a colorless solid (178 mg, 95%). Mp: 136-138 °C; $[\alpha]_D$ –75.0 (*c* 0.4, $CHCl_3$); IR (neat): 2938, 2891, 1642, 1471, 1319, 1162, 1075, 841, 775 cm^{-1}; ^1H NMR ($CDCl_3$, 400 MHz): δ = 5.66-5.58 (m, 2 H), 5.05 (ddd, *J* = 4.4, 6.4, 10.0 Hz, 1 H), 4.00 (dd, *J*

= 6.4, 8.8 Hz, 1 H), 3.79-3.72 (m, 2 H), 3.66 (d, *J* = 12.0 Hz, 1 H), 2.81 (d, *J* = 7.6 Hz, 2 H), 2.72-2.67 (m, 1 H), 2.24 (dd, *J* = 4.0, 12.4 Hz, 1 H), 1.97 (dd, *J* = 2.4, 12.8 Hz, 1 H), 1.54-1.41 (m, 9 H), 1.32 (brs, 2 H), 1.20 (dd, *J* = 12.0, 23.6 Hz, 1 H), 0.81 (s, 9 H), 0.01- (-0.01) (m, 6 H). ^{13}C NMR ($CDCl_3$, 100 MHz): δ = 167.6, 125.3, 121.9, 109.6, 76.0, 69.5, 67.2, 63.4, 58.4, 43.7, 38.0, 37.4, 34.4, 33.2, 25.7, 25.2, 24.1, 23.7, 18.0, -4.6, -4.7. HRMS (QTOF ES+) found *m/z* 444.2539 $(M+Na)^+$, $C_{23}H_{39}NNaO_4Si$ requires 444.2546.

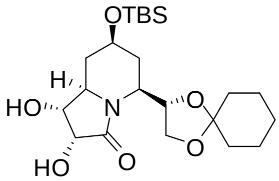

(1*R*,2*R*,5*S*,7*R*,8*aR*)-7-(*tert*-Butyldimethylsilyloxy)-1,2-dihydroxy-5-((*S*)-1,4-dioxaspiro[4.5]decan-2-yl)hexahydroindolizin-3(5*H*)-one (13).

NMO (115 mg, 0.98 mmol) was added in one portion to a solution of the olefin **10** (200 mg, 0.49 mmol) in a mixture of acetone / water (4:1) (5 mL) at room temperature, and then a solution of OsO_4 in water (1 % by wt, 0.7 mL) was added drop wise over 5 minute. The resulting mixture was stirred for 12 h. before being quenched by addition of granular sodium bisulfite (20 mg). The inhomogeneous mixture was then filtered and the filtrate was concentrated in *vacuo* to leave a residue which was extracted with ethyl acetate (2 × 30 mL). The combined organic part was washed with water (2 × 30 mL), brine solution (1 × 30 mL) and then dried over $MgSO_4$. The dried organic solution was then filtered and the filtrate was concentrated under reduced pressure to leave a crude mass which was purified over silica gel using 80 % ethyl acetate-hexane to give a colourless crystalline solid **13** (208 mg, 96 %). Mp: 222-224 °C. $[\alpha]_D$ +9.2 (*c* 0.65, MeOH). IR (neat): 3436, 2934, 2859, 1706, 1686, 1671, 1447, 1374, 1254, 1164, 1124, 1093, 837, 770 cm^{-1}. ^{1}H NMR ($CDCl_3$, 400 MHz): δ = 4.81 (ddd, *J* = 3.2, 6.0, 9.6 Hz, 1 H), 4.31 (br m, 1 H), 4.16 (dd, *J* = 7.0, 9.6 Hz, 2 H), 4.01 (d, *J* = 5.2 Hz, 1 H), 3.9 (dd, *J* = 3.2, 9.2 Hz, 1 H), 3.74-3.67 (m, 1 H), 3.26 (dd, *J* = 2.4, 13.2 Hz, 1 H), 2.99 (s, 1 H), 2.95-2.89 (m, 1 H), 2.23 (br d, *J* = 12.4 Hz, 1 H), 1.97 (br d, *J* = 12.0 Hz, 1 H), 1.62-1.50 (m, 8 H), 1.33 (brm, 2 H), 1.21-1.03 (m, 2 H), 0.80 (s, 9 H), -0.01 (s, 6 H). ^{13}C NMR ($CDCl_3$, 100 MHz): δ = 171.2, 110.0, 74.7, 70.7, 69.3, 69.1, 68.3, 62.9, 59.0, 38.9, 38.4, 37.5, 34.3, 25.7, 25.2, 24.1, 23.7, 18.0, -4.67, -4.7. HRMS (QTOF ES+) found *m/z* 464.2445 $(M + Na)^+$; $C_{22}H_{39}NNaO_6Si$ requires 464.2444.

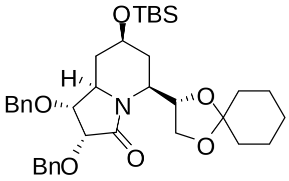

(1*R*,2*R*,5*S*,7*S*,8*aR*)-1,2-Bis(benzyloxy)-7-(*tert*-butyldimethylsilyloxy)-5-((*S*)-1,4-dioxaspiro[4.5]decan-2-yl)hexahydroindolizin-3(5*H*)-one (14).

A solution of the diol **13** (100 mg, 0.23 mmol) in anhydrous THF (2 mL) was added drop wise to a stirred suspension of sodium hydride (28.0 mg, 0.64 mmol) in THF (3 mL) at 0 °C under nitrogen atmosphere. The reaction mixture was then allowed to come to room temperature and stirred for 10 minutes. The resulting solution was cooled back to 0 °C and benzyl bromide (110 μL, 0.92 mmol) followed by *n*-tetrabutylammonium iodide (cat. 4 mg) were sequentially added. The reaction mixture was stirred for 6 h at room temperature, quenched with saturated NH_4Cl solution (2 ml) at 0 °C, and then extracted with ethyl acetate (2 × 20 mL). The combined organic layer was washed sequentially with water (1 × 20 mL), brine (1 × 20 mL), dried over anhydrous Na_2SO_4 and then filtered. The filtrate was concentrated under reduced pressure to leave a crude mass which was purified by column chromatography over silica gel using ethyl acetate in petroleum ether (1:9) to provide the compound **14** (98 mg, 70 %) as a colorless oil. $[\alpha]_D$ + 26.1 (*c* 0.88, $CHCl_3$); IR ($CHCl_3$): 2931, 2856, 1701, 1450, 1254, 1123, 1112, 837, 776 cm^{-1}; ^1H NMR ($CDCl_3$, 400 MHz): δ = 7.31-7.19 (m, 10 H), 4.95 (ddd, *J* = 3.2, 6.0, 9.6 Hz, 1 H), 4.90 (d, *J* = 12.0 Hz, 1 H), 4.79 (d, *J* = 12.0 Hz, 1 H), 4.69 (d, *J* = 12 Hz, 1 H), 4.55 (d, *J* = 12.0 Hz, 1 H), 4.28 (dd, *J* = 6.0, 9.6 Hz, 1 H), 4.04 (dd, *J* = 3.2, 9.6 Hz, 1 H), 3.96 (d, *J* = 5.6 Hz, 1 H), 3.75 (dd, *J* = 2.4, 6.0 Hz, 1 H), 3.72-3.68 (m, 1 H), 3.37-3.34 (m, 1 H), 3.00-2.94 (m, 1 H), 2.28-2.24 (m, 1 H), 1.99-1.95 (m, 1 H), 1.66-1.57 (m, 8 H), 1.42-1.33 (m, 2 H), 1.22 (dd, *J* = 12.0, 24.0 Hz, 1 H), 1.05 (dd, *J* = 12.4, 23.2 Hz, 1 H), 0.88 (s, 9 H), 0.08-0.05 (m, 6 H); ^{13}C NMR ($CDCl_3$, 100 MHz): δ = 169.5, 137.5, 137.4, 128.6, 128.5, 128.4, 128.2, 128.0, 127.0, 109.8, 76.3, 75.6, 74.8, 72.5, 72.2, 69.0, 68.6, 60.5, 58.9, 39.3, 38.3, 37.5, 34.2, 25.8, 25.2, 24.1, 23.7, 18.1, -4.6, -4.7. HRMS (QTOF ES+) found *m/z* 644.3394 $(M + Na)^+$, $C_{36}H_{51}NNaO_6Si$ requires 644.3383.

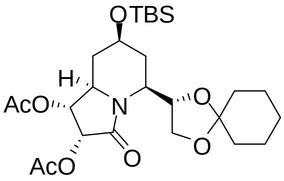

(1*R*,2*R*,5*S*,7*S*,8*aR*)-7-(*tert*-Butyldimethylsilyloxy)-3-oxo-5-((*S*)-1,4-dioxaspiro[4.5]decan-2-yl)octahydroindolizine-1,2-diyl diacetate (15).

Acetic anhydride (120 mg, 1.15 mmol) was added to a stirred solution of the diol **13** (100 mg, 0.23 mmol) in anhydrous pyridine (2 mL) and the resulting reaction mixture was stirred for 12 h at room temperature. The reaction mixture was then diluted with ethyl acetate (1 × 50 mL) and the organic extract was washed sequentially with HCl (1 N, 10 mL), water (1 × 20 mL) followed by brine solution (1 × 20 mL). The organic part was then dried over anhydrous Na_2SO_4, filtered and the filtrate was concentrated under reduced pressure to leave a crude mass which was purified over silica gel using ethyl acetate/petroleum ether (2:9) to provide the compound **15** (95 mg, 80 %) as a colorless solid. $[\alpha]_D$ –7.6 (*c* 0.42, $CHCl_3$); ^{1}H NMR ($CDCl_3$, 400 MHz): δ = 5.42 (d, *J* = 6.0 Hz, 1 H), 5.23 (d, *J* = 6.0 Hz, 1 H), 4.96 (ddd, *J* = 2.8, 6.4, 9.6 Hz, 1 H), 4.28 (dd, *J* = 6.0, 9.2 Hz, 1 H), 4.06 (dd, *J* = 2.8, 9.2 Hz, 1 H), 3.78 (ddd, *J* = 4.4, 10.4, 15.2 Hz, 1 H), 3.36 (dd, *J* = 2.8, 13.2 Hz, 1 H), 3.00 (dt, *J* = 2.4, 10.8 Hz, 1 H), 2.35-2.31 (m, 1 H), 2.22-2.18 (m, 1 H), 2.15 (s, 3 H), 2.11 (s, 3 H), 1.64-1.58 (m, 8 H), 1.47-1.42 (m, 2 H), 1.35-1.30 (m, 2 H), 0.96 (s, 9 H), 0.01 (s, 6 H); ^{13}C NMR ($CDCl_3$, 100 MHz): δ = 170.0 (s), 169.6 (s), 166.3 (s), 110.1 (s), 74.7 (d), 70.0 (d), 69.1 (d), 68.8 (d), 68.2 (t), 60.8 (d), 59.1 (d), 38.6 (t), 38.2 (t), 37.6 (t), 34.2 (t), 25.7 (q), 25.1 (t), 24.1 (t), 23.7 (t), 20.6 (q), 20.3 (q), 18.0 (s), -4.6 (q), -4.7 (q); HRMS (QTOF ES+) found *m/z* 548.2661 (M + Na)$^+$, $C_{26}H_{43}NNaO_8Si$ requires 548.2656.

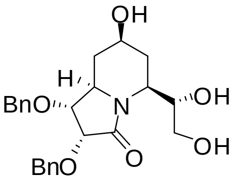

(1*R*,2*R*,5*S*,7*S*,8*aR*)-1,2-Bis(benzyloxy)-5-((*S*)-1,2-dihydroxyethyl)-7-hydroxyhexahydroindolizin-3(5*H*)-one (16).

HCl (10 %, 2 mL) was added drop wise to a stirred solution of the acetal derivative **14** (60 mg, 0.09 mmol) in THF (2 mL) at 0 °C. The resulting mixture was allowed to come to room temperature and stirred for 18 h. The homogeneous solution was then diluted with water (2

mL) and neutralized with solid $NaHCO_3$ before being extracted with ethyl acetate (2 × 10 mL). The combined organic extract was washed successively with water (1 × 5 mL), brine solution (1 × 5 mL), dried over $MgSO_4$ and then filtered. The filtrate was concentrated in *vacuo* to leave a crude viscous liquid which was purified by flash chromatography over silica gel using 20 % methanol in ethyl acetate to provide **16** (36 mg, 89 %) as a colorless gum. $[\alpha]_D$ +5.1 (*c* 0.88, $CHCl_3$); IR ($CHCl_3$): 3438, 2935, 2862, 1706, 1684, 1671, 1374, 1258, 1161, 1124, 774 cm^{-1}; ^1H NMR ($CDCl_3$, 400 MHz): δ = 7.41-7.11 (m, 10 H), 5.00 (brs, 1 H), 4.92 (d, *J* = 12.0 Hz, 1 H), 4.77 (d, *J* = 12.0 Hz, 1 H), 4.66 (d, *J* = 12.0 Hz, 1 H), 4.51 (d, *J* = 12.0 Hz, 1 H), 4.44 (brs, 1 H), 4.04 (d, *J* = 5.6 Hz, 1 H), 3.82 (brm, 1 H), 3.73 (dd, *J* = 2.8, 5.2 Hz, 1 H), 3.70-3.66 (m, 1 H), 3.59 (brm, 1 H), 3.50-3.47 (m, 1 H), 3.26 (d, *J* = 11.2 Hz, 1 H), 2.83 (brs, 1 H), 2.60 (brs, 1 H), 2.20-2.17 (m, 1 H), 1.97-1.93 (m, 1 H), 1.50 (dd, *J* = 11.2, 22.8 Hz, 1 H), 1.04 (dd, *J* = 12.0, 23.2 Hz, 1 H); ^{13}C NMR ($CDCl_3$, 100 MHz): δ = 170.6, 137.3, 128.54, 128.5, 128.2, 128.1, 128.0, 127.7, 76.4, 75.9, 72.7, 72.2, 69.7, 67.5, 62.8, 61.1, 57.7, 33.7, 29.7. HRMS (QTOF ES+) found *m/z* 450.1892 $(M + Na)^+$; $C_{24}H_{29}NNaO_6$ requires 450.1893.

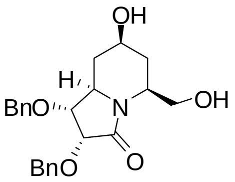

(1*R*,2*R*,5*S*,7*S*,8*aR*)-1,2-Bis(benzyloxy)-7-hydroxy-5-(hydroxymethyl)hexahydroindolizin-3(5*H*)-one (17).

$NaIO_4$ (80 mg, 0.38 mmol) was added portion wise over 5 min to a solution of the diol **16** (80 mg, 0.19 mmol) in acetonitrile /water (v/v 3:1) (3 mL) at 5-10 °C and the resulting mixture was stirred for 30 min. The reaction mixture was allowed to come to room temperature and then filtered. The filtrate was diluted with dichloromethane (20 mL) and the combined organic solution was washed sequentially with water (1 × 10 mL), brine solution (1 × 10 mL) and then dried over $MgSO_4$. The organic part was filtered and the filtrate was concentrated in *vacuo* to leave the crude product which was used as such in the next step. The crude aldehyde, thus obtained, was dissolved in dry methanol (2 mL) and cooled to 0 °C. $NaBH_4$ (10 mg, 0.28 mmol) was then added in one portion and the resulting reaction mixture was allowed to come to room temperature over 30 min while stirring. The reaction mixture was then concentrated and the residue was extracted with ethyl acetate (2 × 10 mL). The combined organic extract was washed with water (5 mL), brine solution (5 mL), and dried

over $MgSO_4$. The organic part was then filtered and the filtrate was concentrated in *vacuo* to leave a crude product which was purified over silica gel using 50 % ethyl acetate-hexane solution to give the product **17** as a viscous liquid (68 mg, 92 % over two steps). $[\alpha]_D$ +70.1 (*c* 1.5, $CHCl_3$); IR ($CHCl_3$): 3432, 2941, 2859, 1709, 1680, 1371, 1164, 1114, 1094, 774 cm^{-1}; ^1H NMR ($CDCl_3$, 400 MHz): δ = 7.42-7.27 (m, 10 H), 5.02 (br s, 1 H), 4.93 (d, *J* = 11.6 Hz, 1 H), 4.77 (d, *J* = 11.6 Hz, 1 H), 4.65 (d, *J* = 11.6 Hz, 1 H), 4.44 (d, *J* = 11.6 Hz, 1 H), 3.98 (d, *J* = 6.0 Hz, 1 H), 3.90-3.74 (m, 4 H), 3.68 (t, *J* = 5.6 Hz, 1 H), 3.50 (ddd, *J* = 3.2, 4.8, 8.0 Hz, 1 H), 3.23 (ddd, *J* = 2.8, 6.4, 9.2 Hz, 1 H), 2.28-2.24 (m, 1 H), 1.86-1.80 (m, 1 H), 1.02 (dd, *J* = 12.0, 23.2 Hz, 1 H), 0.89-0.84 (m, 1 H); ^{13}C NMR ($CDCl_3$, 100 MHz): δ = 170.7, 137.2, 137.16, 128.56, 128.50, 128.4, 128.15, 128.09, 128.0, 75.0, 72.4, 72.1, 67.4, 63.1, 60.7, 58.1, 38.8, 36.9, 29.7. HRMS (QTOF ES+) found *m/z* 420.1790 $(M + Na)^+$; $C_{23}H_{27}NNaO_5$ requires 420.1787.

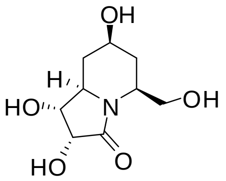

(1*R*,2*R*,5*S*,7*S*,8*aR*)-1,2,7-Trihydroxy-5-(hydroxymethyl)hexahydroindolizin-3(5*H*)-one (18).

$Pd(OH)_2$-C (10%, 4 mg) was added to a solution of the benzyl ether **17** (25 mg, 0.06 mmol) in methanol (2 mL) and the resulting heterogeneous mixture was vigorously stirred under hydrogen atmosphere for 3 h. The reaction mixture was then filtered through celite, the filter cake was washed with methanol (10 mL), and the combined filtrate was concentrated in *vacuo* to leave a crude product which was purified by chromatography over neutral alumina using Meoh/EtOAc (1:4) to obtain the tetrahydroxyindolizidine derivative **18** (11 mg, 81%) as a colourless foam. $[\alpha]_D$ +2.1 (*c* 0.47, MeOH); ^1H NMR (DMSO-d_6, 400 MHz): δ = 5.49 (br s, 1 H), 5.02 (s, 1 H), 4.91-4.88 (m, 1 H), 4.06 (s, 1 H), 3.79 (brm, 3 H), 3.64 (m, 1 H), 3.21-3.07 (m, 3 H), 1.99 (d, *J* = 10.4 Hz, 1 H), 1.79 (d, *J* = 10.8 Hz, 1 H), 1.09-0.96 (m, 2 H). ^{13}C NMR (DMSO-d_6, 100 MHz): δ = 172.5, 71.2, 70.1, 67.0, 63.1, 62.2, 57.7, 38.4, 37.7; HRMS (QTOF ES+) found *m/z* 240.0849 $(M+Na)^+$; $C_9H_{15}NNaO_5$ requires 240.0848.

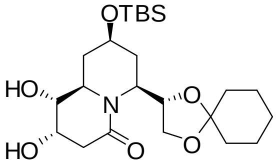

(1*R*,2*S*,6*S*,8*R*,9a*R*)-8-(*tert*-Butyldimethylsilyloxy)-1,2-dihydroxy-6-((*S*)-1,4-dioxaspiro[4.5]decan-2-yl)hexahydro-1*H*-quinolizin-4(6*H*)-one (19).

In a one-necked round bottomed flask equipped with a magnetic stirring bar, olefin **12** (200 mg, 0.49 mmol) was taken in acetone / water (4:1) (5 mL) at room temperature. Solid *N*-methylmorpholine-*N*-oxide (115 mg, 0.98 mmol) followed by an aqueous solution of OsO_4 (1% by w/v, 0.4 mL) were then sequentially added to the reaction mixture which was stirred for 6 h before being quenched with granular sodium bisulfite (200 mg). The reaction mixture was then filtered, the filtrate was concentrated in *vacuo*, and the resulting residue was diluted with ethyl acetate (2 × 30 mL). The combined organic part was washed sequentially with water (2×20 mL) and brine solution (1×20 mL), and then dried over $MgSO_4$. The organic extract was then filtered and the filtrate was concentrated under reduced pressure to leave a crude product which was purified by column chromatography over silica gel using 80 % ethyl acetate-hexane to give compound **19** as a colourless crystalline solid (205 mg, 95 %). Mp: 208 °C; $[\alpha]_D$ +5.1 (*c* 0.6, MeOH); IR (neat): 3466, 3350, 2887, 2859, 1649, 1619, 1123, 1084, 838, 776 cm^{-1}; ^{1}H NMR ($CDCl_3$, 400 MHz): δ = 4.91 (ddd, *J* = 4.4, 6.0, 10.0 Hz, 1 H), 4.04-4.02 (br m, 1 H), 3.94 (dd, *J* = 6.0, 8.0 Hz, 1 H), 3.81-3.72 (m, 3 H), 3.29-3.26 (m, 1 H), 3.11 (d, *J* = 3.2 Hz, 1 H), 3.00 (d, *J* = 5.2 Hz, 1 H), 2.80 (t, *J* = 9.2 Hz, 1 H), 2.54 (dd, *J* = 9.6 Hz, 16.4 Hz, 1 H), 2.42 (dd, *J* = 5.6, 16.4 Hz, 1 H), 2.18 (dd, *J* = 4.4, 12.4 Hz, 1 H), 1.97 (dd, *J* = 4.8, 12.4 Hz, 1 H), 1.52-1.39 (m, 9 H), 1.37-1.30 (m, 2 H), 1.23-1.14 (m, 1 H), 0.8 (s, 9 H), -0.001 (s, 3 H), -0.01 (s, 3 H); ^{13}C NMR ($CDCl_3$, 100 MHz): δ = 168.1 (s), 109.8 (s), 76.1 (d), 70.9 (d), 69.0 (d), 67.0 (t), 65.0 (d), 62.3 (d), 60.4 (d), 40.5 (t), 37.6 (t), 37.4 (t), 37.3 (t), 34.4 (t), 25.7 (q), 25.2 (t), 24.1 (t), 23.7 (t), 18.0 (t), -4.6 (q), -4.7 (q). HRMS (QTOF ES+) found *m/z* 478.2612 $(M + Na)^+$ $C_{23}H_{41}NNaO_6Si$ requires 478.2601.

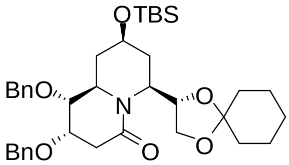

(1*R*,2*S*,6*S*,8*S*,9*aR*)-1,2-Bis(benzyloxy)-8-(*tert*-butyldimethylsilyloxy)-6-((*S*)-1,4-dioxaspiro[4.5]decan-2-yl)hexahydro-1*H*-quinolizin-4(6*H*)-one (20).

Compound **20** was prepared following the procedure described for **14** Yield: 114 mg, 82 %. $[\alpha]_D$ + 71.0 (*c* 0.53, $CHCl_3$); IR ($CHCl_3$): 2931, 2856, 1701, 1450, 1254, 1123, 1112, 837, 776 cm^{-1}; ^1H NMR ($CDCl_3$, 400 MHz): δ = 7.31-7.23 (m, 10 H), 4.96 (ddd, *J* = 4.4, 6.0, 10.0 Hz, 1 H), 4.72 (d, *J* = 12.0 Hz, 1 H), 4.54-4.5 (m, 2 H), 4.46 (d, *J* = 12.0 Hz, 1 H), 3.99 (dd, *J* = 6.4, 8.8 Hz, 1 H), 3.82 (ddd, *J* = 2.4, 4.8, 7.2 Hz, 1 H), 3.75 (dd, *J* = 4.4, 9.2 Hz, 1 H), 3.70 (dd, *J* = 5.2, 10.0 Hz, 1 H), 3.53 (m, 1 H), 3.29-3.26 (m, 1 H), 2.80-2.68 (m, 2 H), 2.41 (dd, *J* = 4.8, 16.4 Hz, 1 H), 2.19 (dd, *J* = 4.8, 12.4 Hz, 1 H), 1.87 (dd, *J* = 4.8, 12.4 Hz, 1 H), 1.55-1.51 (m, 10 H), 1.42-1.35 (m, 1 H), 1.14-1.05 (m, 1 H), 0.81(s, 9 H), -0.001 (s, 3 H), -0.01 (s, 3 H); ^{13}C NMR ($CDCl_3$, 100 MHz): δ = 167.4, 138.1, 137.8, 128.5, 127.9, 127.8, 127.6, 109.5, 76.6, 76.0, 72.3, 71.1, 71.0, 69.2, 67.4, 62.7, 58.9, 41.0, 37.9, 37.2, 35.5, 34.4, 25.8, 25.3, 24.1, 23.8, 18.0, -4.6, -4.7. HRMS (QTOF ES+) found *m/z* 658.3530 $(M + Na)^+$, $C_{37}H_{53}NNaO_6Si$ requires 658.3540.

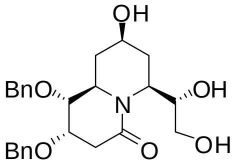

(1*R*,2*S*,6*S*,8*S*,9*aR*)-1,2-Bis(benzyloxy)-6-((*S*)-1,2-dihydroxyethyl)-8-hydroxyhexahydro-1*H*-quinolizin-4(6*H*)-one (21).

Compound **21** was prepared following the procedure described for **16** but using 12 h instead of 18 h. Yield: 33 mg, 80%.$[\alpha]_D$ + 16.6 (*c* 1.1, $CHCl_3$); IR ($CHCl_3$): 3438, 2933, 2860, 1706, 1679, 1374, 1256, 1167, 1121, 776 cm^{-1};^1H NMR ($CDCl_3$, 400 MHz): δ = 7.38-7.29 (m, 10 H), 4.65 (d, *J* = 12.0 Hz, 1 H), 4.64 (d, *J* = 11.6 Hz, 1 H), 4.56 (d, *J* = 11.6 Hz, 1 H), 4.48 (d, *J* = 11.6 Hz, 1 H), 4.11-4.03 (m, 2 H), 3.97 (br s, 1 H), 3.70 (d, *J* = 4.4 Hz, 1 H), 3.65-3.49 (m, 3 H), 3.47 (dd, *J* = 1.6, 7.2 Hz, 1 H), 2.84 (dd, *J* = 5.2, 17.2 Hz, 1 H), 2.55-2.48 (m, 2 H), 2.09-2.03 (m, 1 H), 1.93-1.86 (m, 2 H), 1.42-1.30 (m, 1 H), 0.89-0.83 (m, 2 H); ^{13}C NMR ($CDCl_3$, 100 MHz): δ = 169.0, 137.8, 137.6, 128.7, 128.6, 128.5, 128.1, 127.9, 127.8, 77.6, 77.3, 72.1, 71.6, 71.3, 69.8, 65.1, 63.3, 37.0, 36.1, 32.1, 29.7. HRMS (QTOF ES+) found *m/z* 464.2046 $(M + Na)^+$, $C_{25}H_{31}NNaO_6$ requires 464.2049.

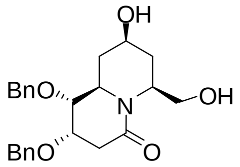

(1*R*,2*S*,6*S*,8*S*,9*aR*)-1,2-Bis(benzyloxy)-8-hydroxy-6-(hydroxymethyl)hexahydro-1*H*-quinolizin-4(6*H*)-one (22).

Compound **22** was prepared following the procedure described for **17**. Yield: 67 mg, 90 %. $[\alpha]_D$ – 68.0 (*c* 3.0, $CHCl_3$); IR ($CHCl_3$): 3468, 3348, 2889, 2859, 1644, 1619, 1123, 1082, 846, 776 cm^{-1}. ^1H NMR ($CDCl_3$, 400 MHz): δ = 7.38-7.28 (m, 10 H), 4.69 (d, *J* = 12.0 Hz, 1 H), 4.64 (d, *J* = 12.0 Hz, 1 H), 4.57 (d, *J* = 12.0 Hz, 1 H), 4.51 (d, *J* = 12.0 Hz, 1 H), 4.03-3.89 (m, 3 H), 3.68 (dd, *J* = 4.8, 12.4 Hz, 1 H), 3.53-3.45 (m, 2 H), 3.41-3.3.38 (m, 1 H), 2.87 (dd, *J* = 7.2, 17.2 Hz, 1 H), 2.52 (dd, *J* = 4.4, 17.2 Hz, 1 H), 2.36 (dd, *J* = 6.8, 12.4 Hz, 1 H), 2.00 (dt, *J* = 4.0, 14.0 Hz, 2 H), 1.70 (dt, *J* = 8.0, 13.6 Hz, 1 H), 1.37-1.20 (m, 2 H). ^{13}C NMR ($CDCl_3$, 100 MHz): δ = 168.3, 137.8, 137.6, 128.5, 128.0, 127.9, 127.7, 72.1, 71.2, 70.0, 66.1, 63.4, 58.9, 56.1, 38.5, 35.8, 35.7, 29.7. HRMS (QTOF ES+) found *m/z* 434.1952 (M + Na)$^+$, $C_{24}H_{29}NNaO_5$ requires 434.1943.

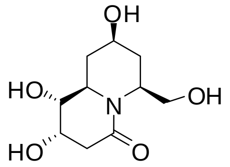

(1*R*,2*S*,6*S*,8*S*,9a*R*)-1,2,8-Trihydroxy-6-(hydroxymethyl)hexahydro-1*H*-quinolizin-4(6*H*)-one (23).

Compound **23** was prepared following the procedure described for **18** but using a time of 6 h. Yield: 11 mg, 80 %. $[\alpha]_D$ – 31.0 (*c* 0.95, MeOH). ^1H NMR (DMSO-d_6, 400 MHz): δ = 5.07 (d, *J* = 5.2 Hz, 1 H), 4.94 (d, *J* = 3.6 Hz, 1 H), 4.91 (d, *J* = 4.0 Hz, 1 H), 4.79 (t, *J* = 6.0 Hz, 1 H), 3.8 (br m, 3 H), 3.72-3.66 (m, 2 H), 3.22 (dd, *J* = 7.6, 12.4 Hz, 1 H), 3.16 (d, *J* = 4.8 Hz, 1 H), 2.39 (dd, *J* = 4.0, 16.8 Hz, 1 H), 2.31 (dd, *J* = 6.4, 16.8 Hz, 1 H), 2.21 (dd, *J* = 6.4, 12.8 Hz, 1 H), 1.98-1.86 (m, 1 H), 1.56-1.51 (m, 1 H), 1.16-1.10 (m, 1 H). ^1H NMR (D_2O, 600 MHz): δ = 4.20-4.18 (m, 1 H), 4.13-4.10 (m, 1 H), 3.92 (dd, *J* = 5.4, 12.0 Hz, 1 H), 3.87 (dd, *J* = 5.4, 12.0 Hz, 1 H), 3.84 (dd, *J* = 1.8, 5.4 Hz, 1 H), 3.56-3.54 (m, 1 H), 3.48 (dd, *J* = 4.2, 12.0 Hz, 1 H), 2.68 (dd, *J* = 4.8, 17.4 Hz, 1 H), 2.59 (dd, *J* = 6.6, 17.4 Hz, 1 H), 2.38 (q, *J* = 6.0 Hz, 1 H), 2.09-2.07 (m, 1 H), 1.68 (dt, *J* = 7.8, 13.8 Hz, 1 H), 1.38 (dd, *J* = 12.0, 22.2 Hz, 1 H). ^{13}C NMR (DMSO-d_6, 100 MHz): δ = 168.3, 71.0, 65.6, 65.4, 63.1, 58.1, 56.9, 38.9, 38.7, 35.1. HRMS (QTOF ES+) found *m/z* 254.1008 (M + Na)$^+$, $C_{10}H_{17}NNaO_5$ requires 254.1004.

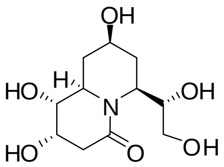

(1*R*,2*S*,6*S*,8*S*,9*aR*)-6-((*S*)-1,2-Dihydroxyethyl)-1,2,8-trihydroxyhexahydro-1*H*-quinolizin-4(6*H*)-one (24).

Compound **24** was prepared following the procedure described for **18** but using a time of 6 h. Yield: 25 mg (from 51 mg, 85 %). $[\alpha]_D$ – 76.0 (*c* 0.6, MeOH). ^{1}H NMR (D_2O, 400 MHz): δ = 4.22-4.18 (m, 1 H), 4.12-4.09 (m, 1 H), 3.96-3.93 (m, 1 H), 3.78-3.77 (m, 1 H), 3.48 (dd, *J* = 4.8, 12.0 Hz, 1 H), 3.45-3.40 (m, 2 H), 3.22-3.20 (m, 1 H),2.53-2.48 (m, 2 H), 2.19 (dd, *J* = 6.0, 12.4 Hz, 1 H), 2.03-1.99 (m, 1 H), 1.46 (dt, *J* = 9.2, 13.2 Hz, 1 H), 1.29-1.20 (m, 1 H). ^{13}C NMR (D_2O, 100 MHz): δ = 170.9, 70.8, 69.7, 66.7, 64.4, 62.8, 59.6, 59.4, 37.7, 36.5, 32.6. HRMS (QTOF ES+) found *m/z* 284.1104 $(M + Na)^+$, $C_{11}H_{19}NNaO_6$ requires 284.1110.

Copies of NMR spectral data for all new compounds

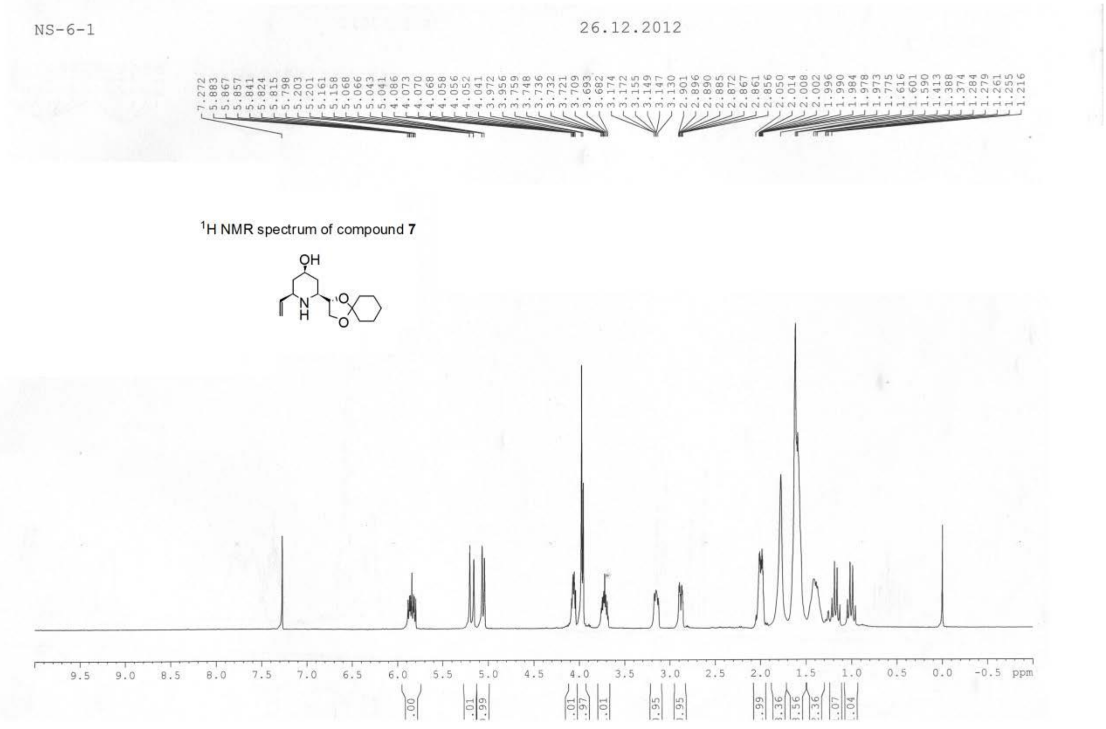

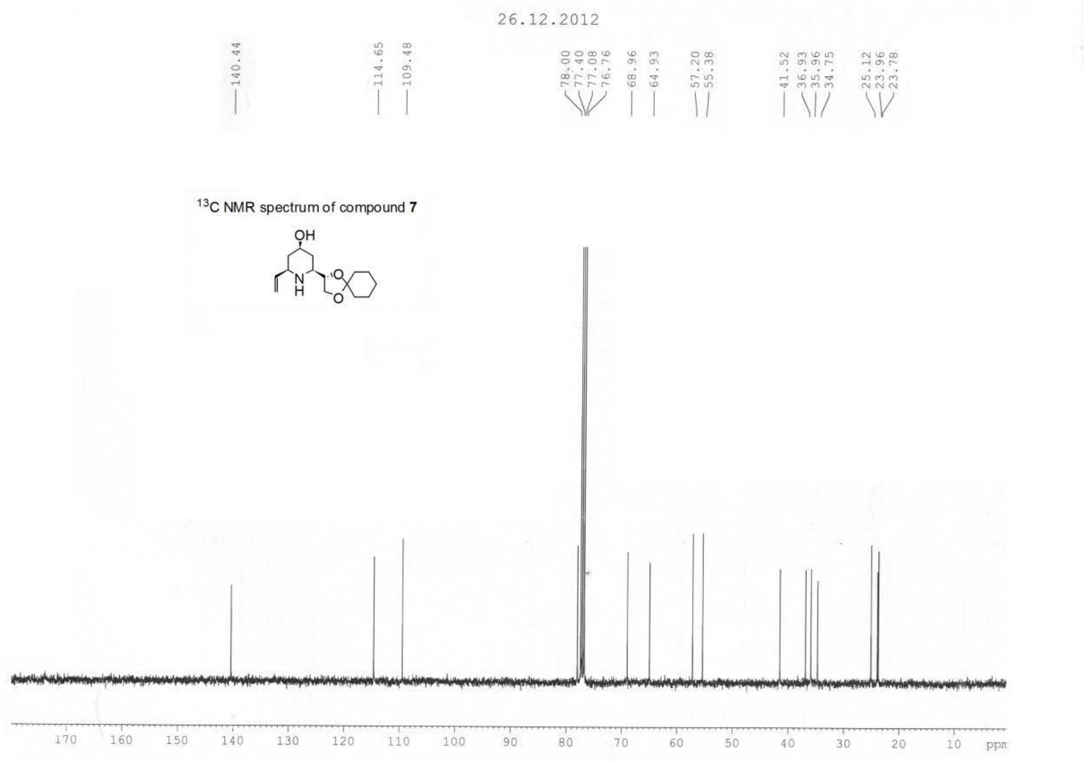

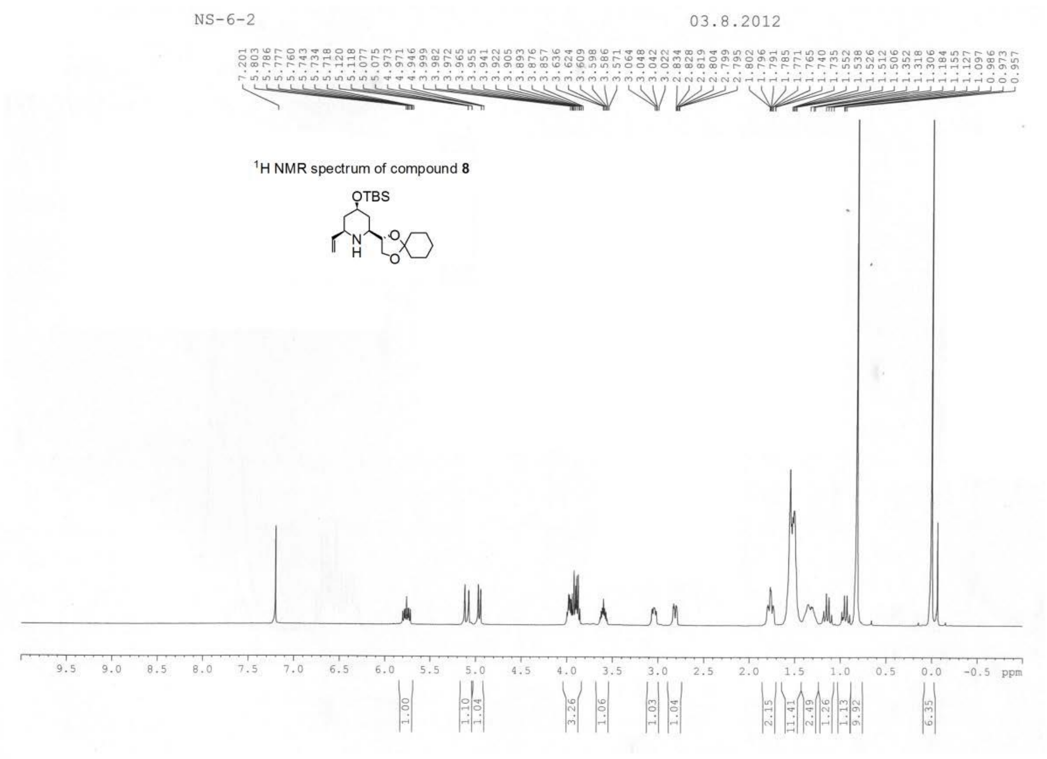
NS-6-2
03.8.2012
1H NMR spectrum of compound 8
OTBS
N
H
O
O
ppm

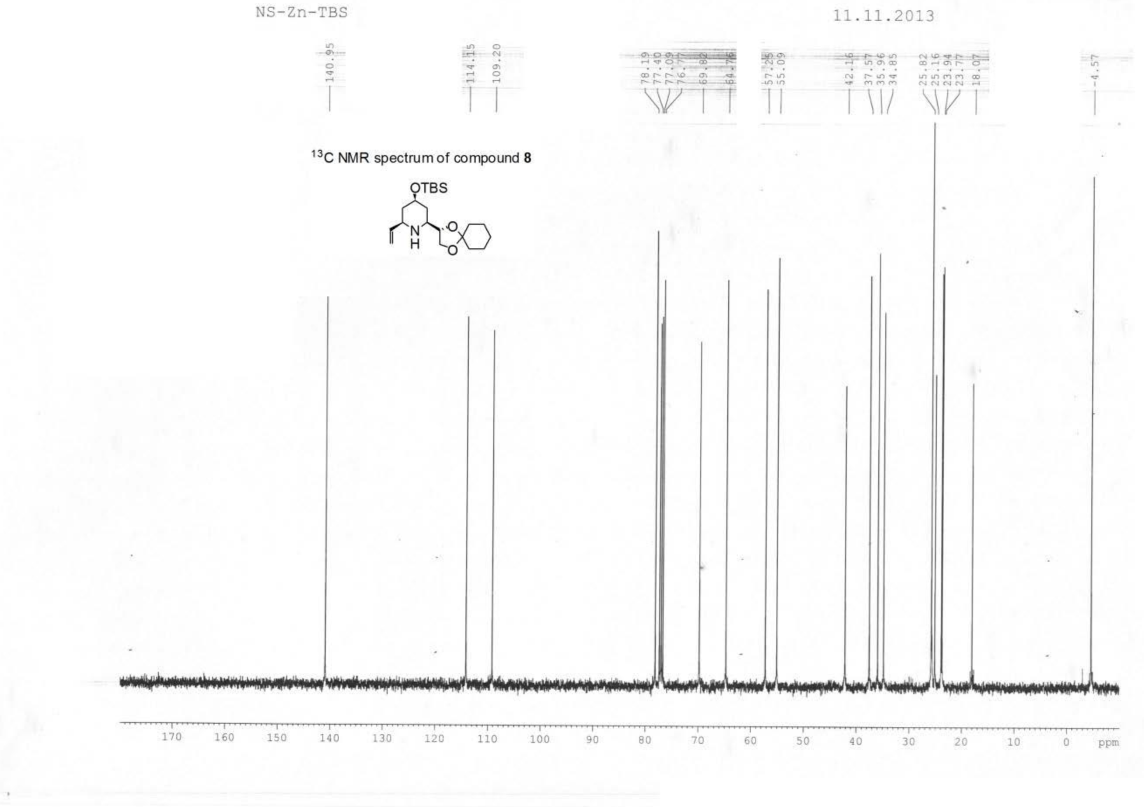
NS-Zn-TBS
11.11.2013
13C NMR spectrum of compound 8
OTBS
N
H
O
O
ppm

^{1}H NMR spectrum of compound **9**

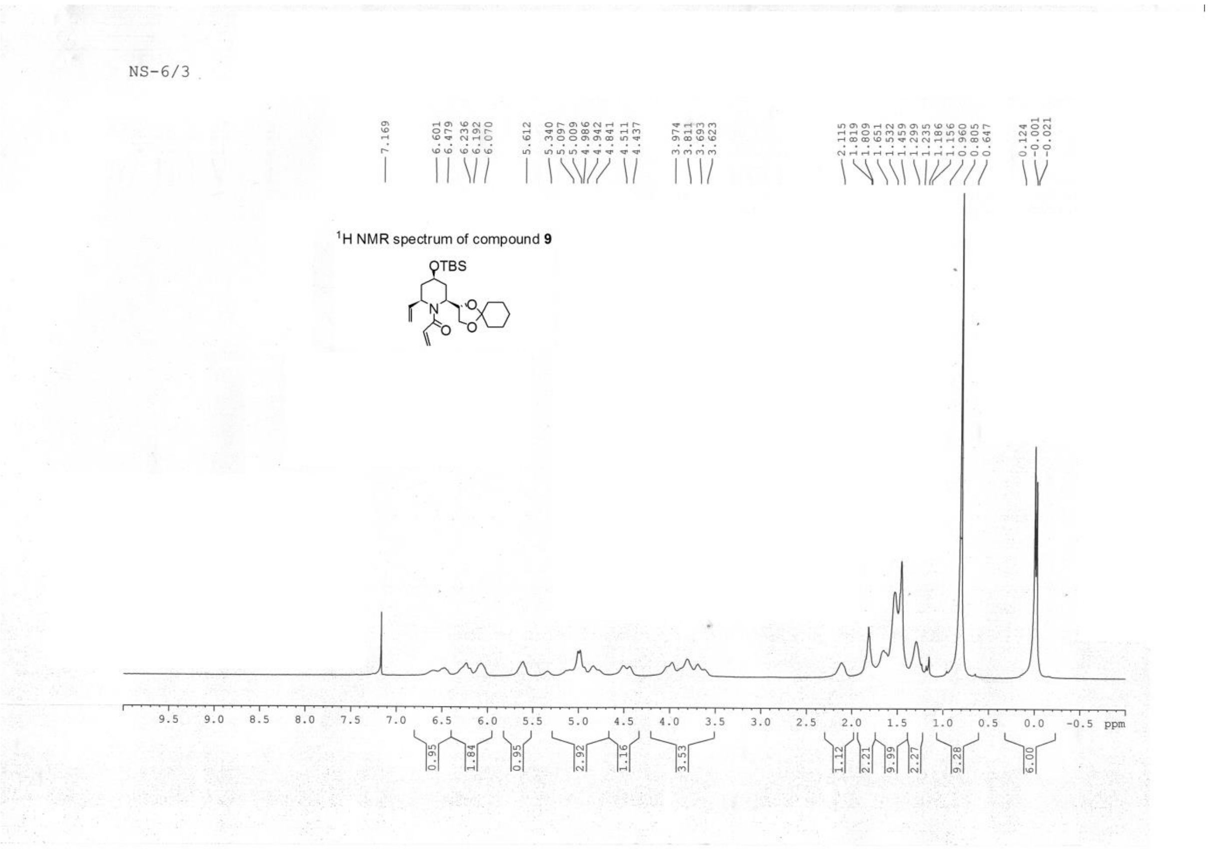

^{13}C NMR spectrum of compound **9**

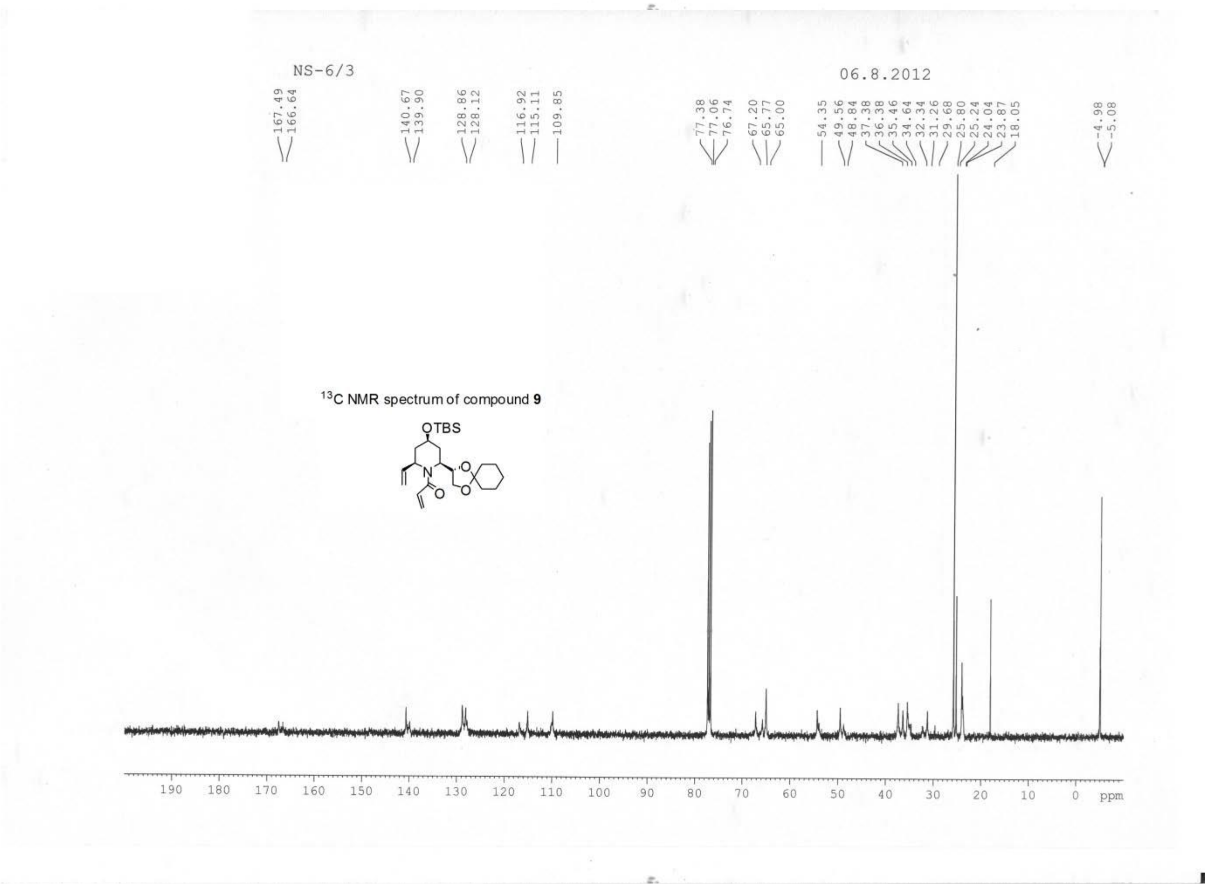

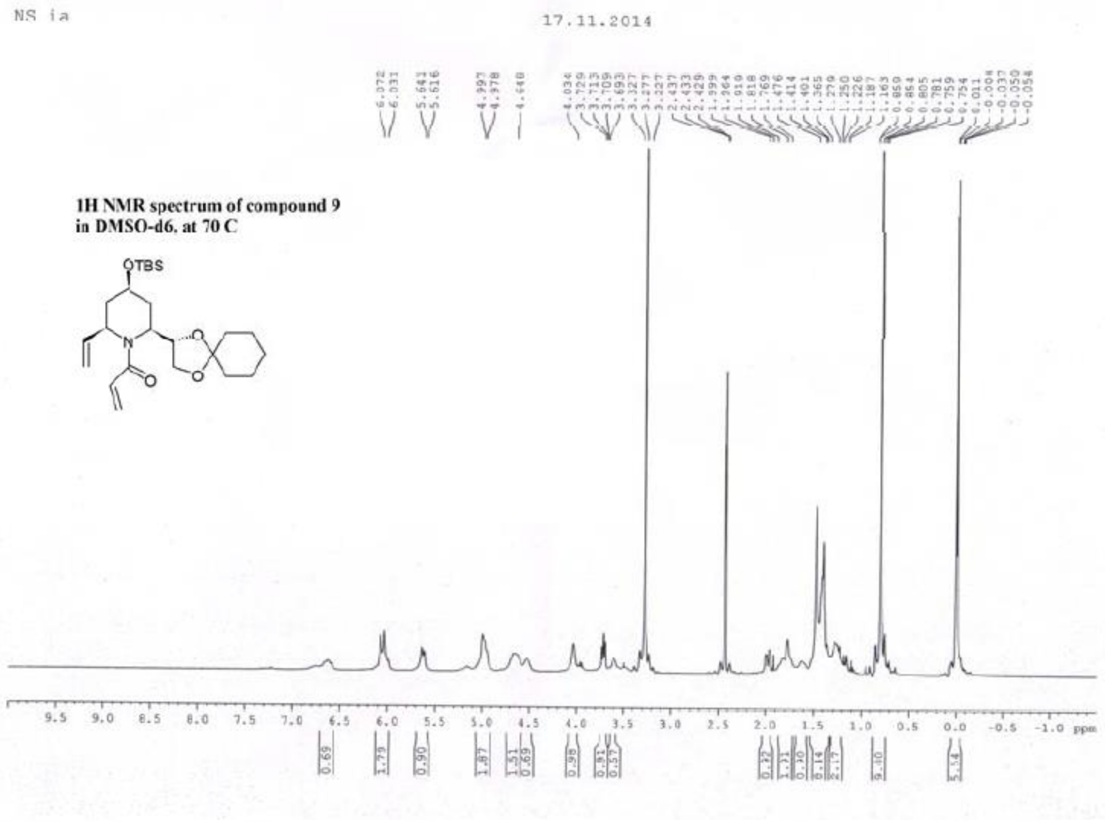
NS ia
17.11.2014
1H NMR spectrum of compound 9
in DMSO-d6, at 70 C
OTBS
N
O
O
O
ppm

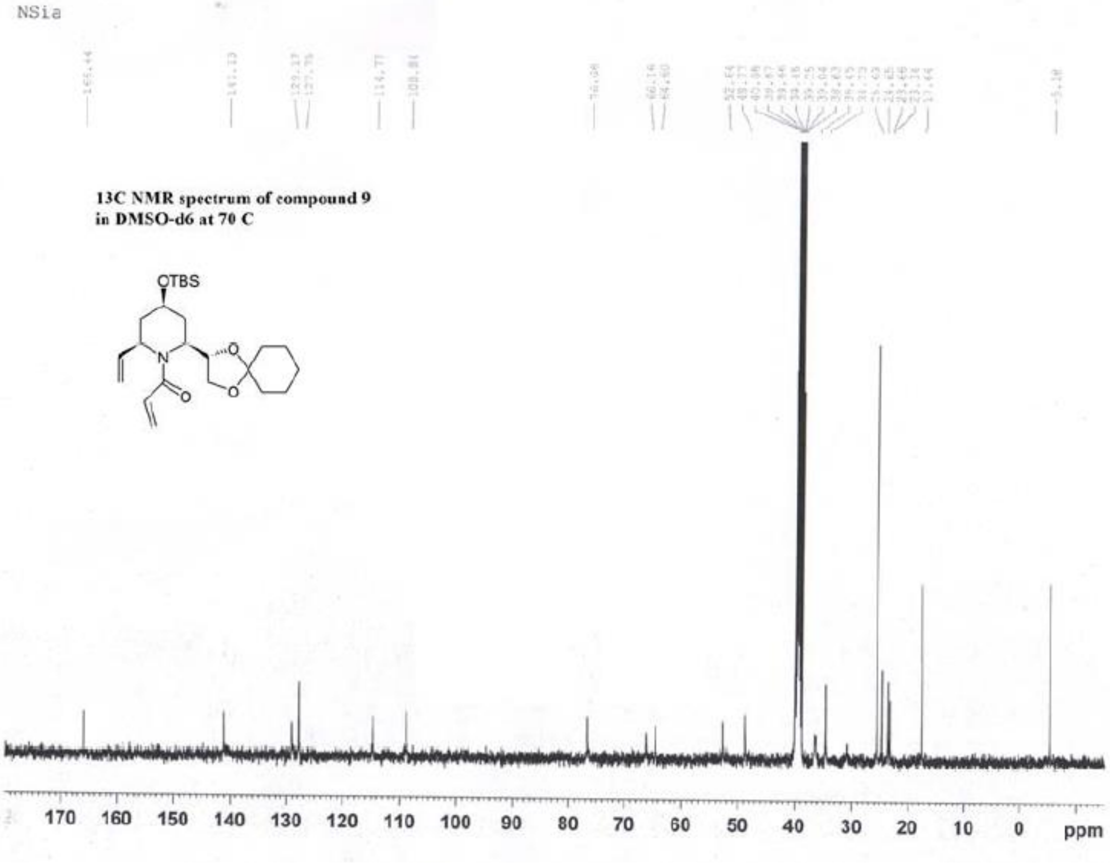
NSia
13C NMR spectrum of compound 9
in DMSO-d6 at 70 C
OTBS
N
O
O
O
ppm

1H NMR spectrum of compound **10**

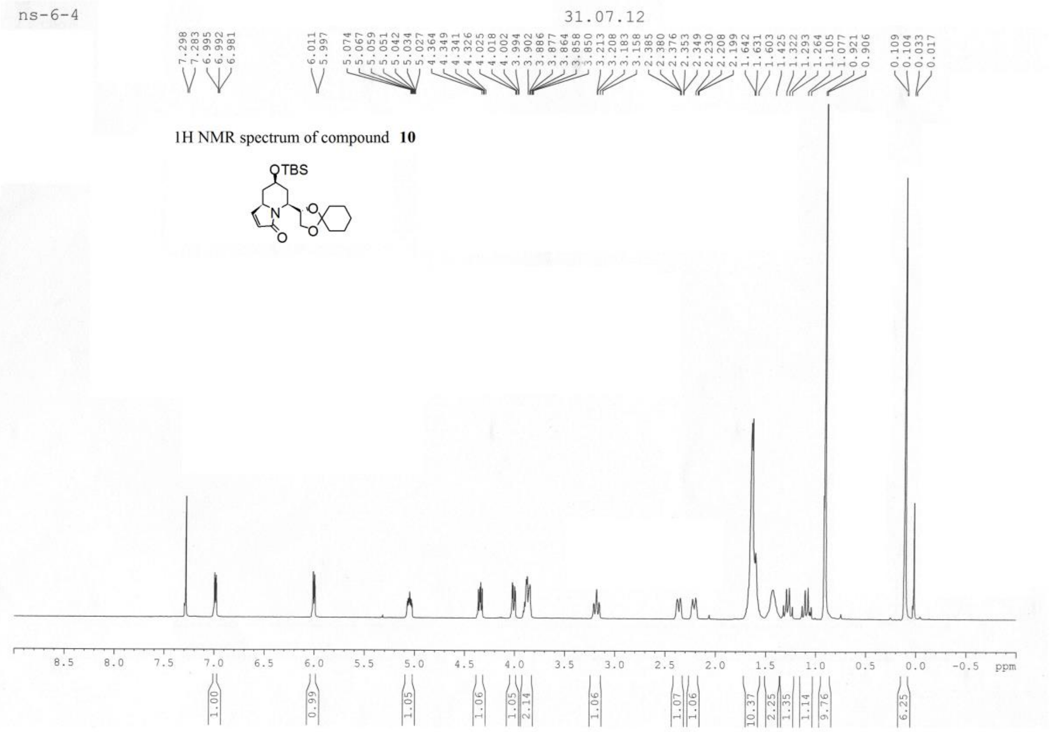

13C NMR spectrum of compound **10**

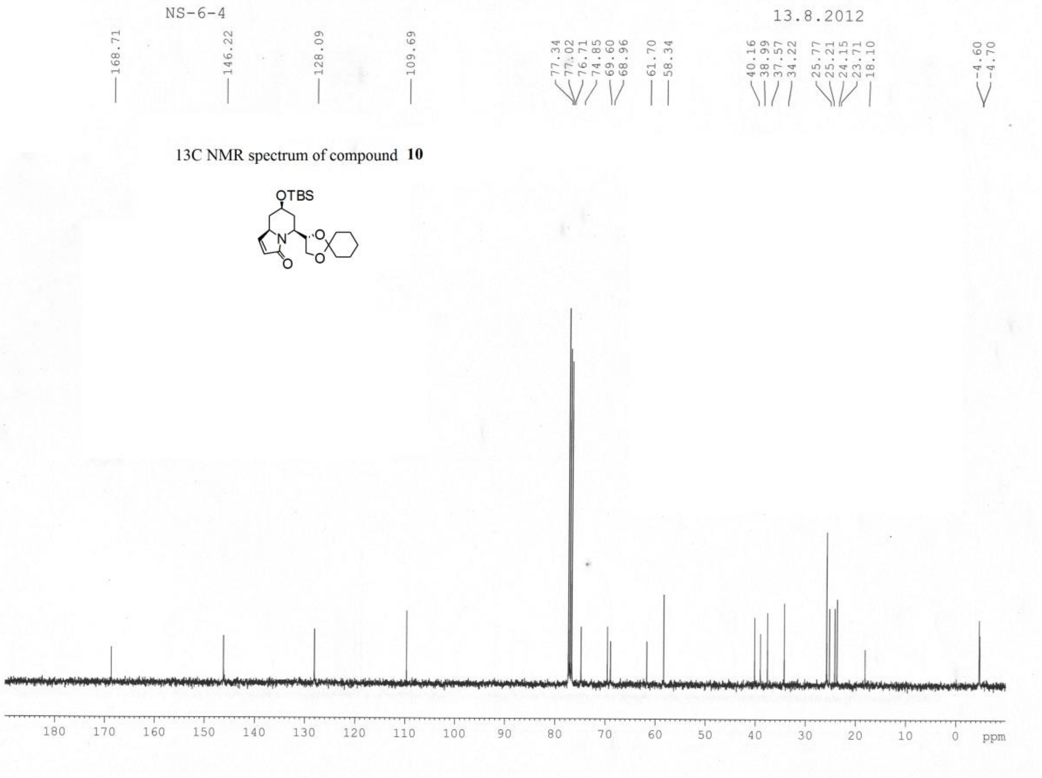

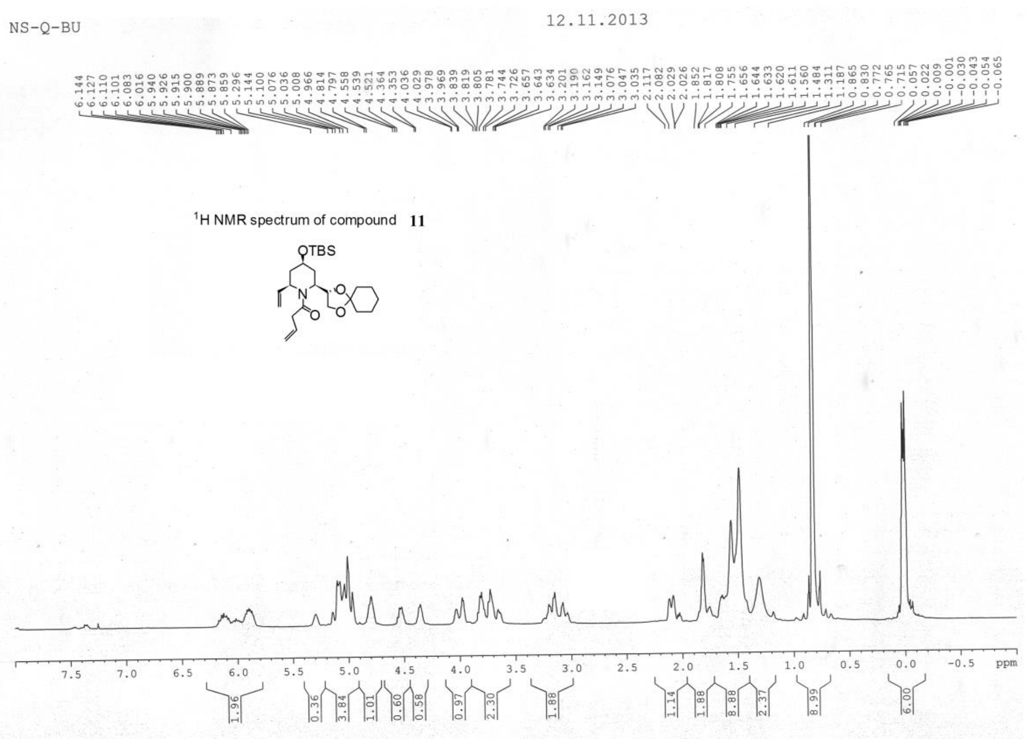
NS-Q-BU
12.11.2013
1H NMR spectrum of compound 11
OTBS
ppm

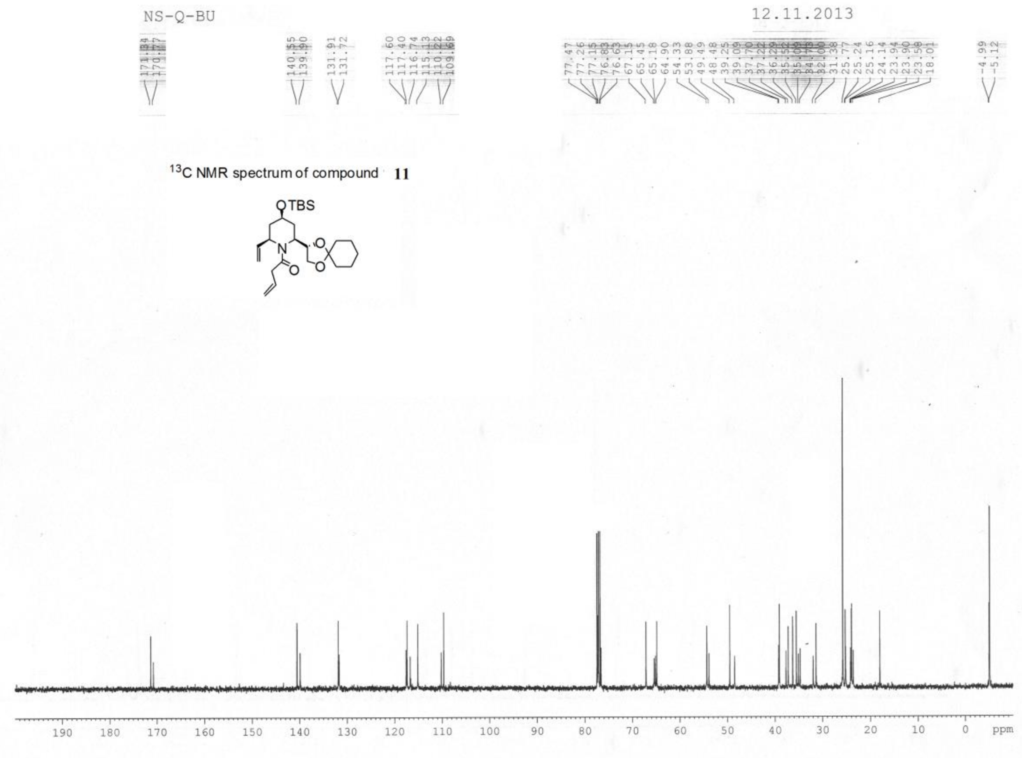
NS-Q-BU
12.11.2013
13C NMR spectrum of compound 11
OTBS
ppm

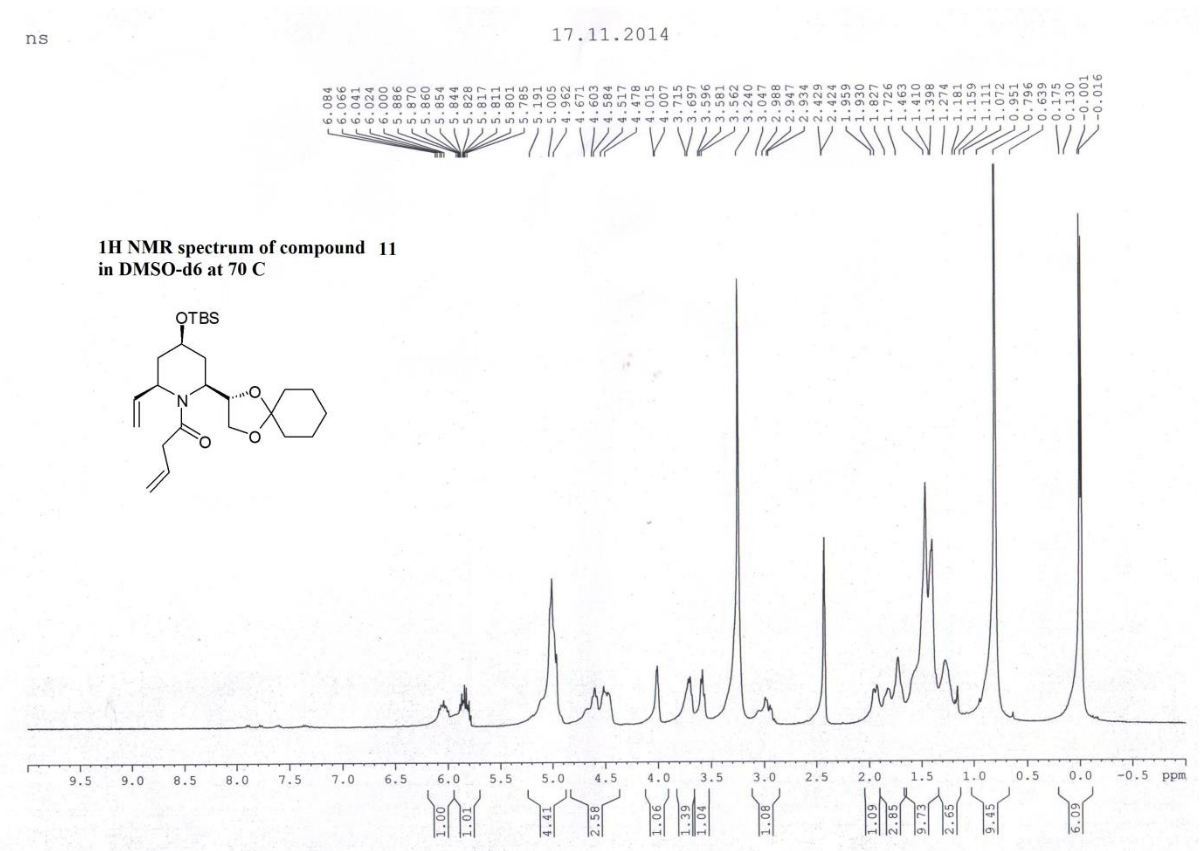
ns
17.11.2014
1H NMR spectrum of compound 11
in DMSO-d6 at 70 C
OTBS
N
O
O
O
ppm

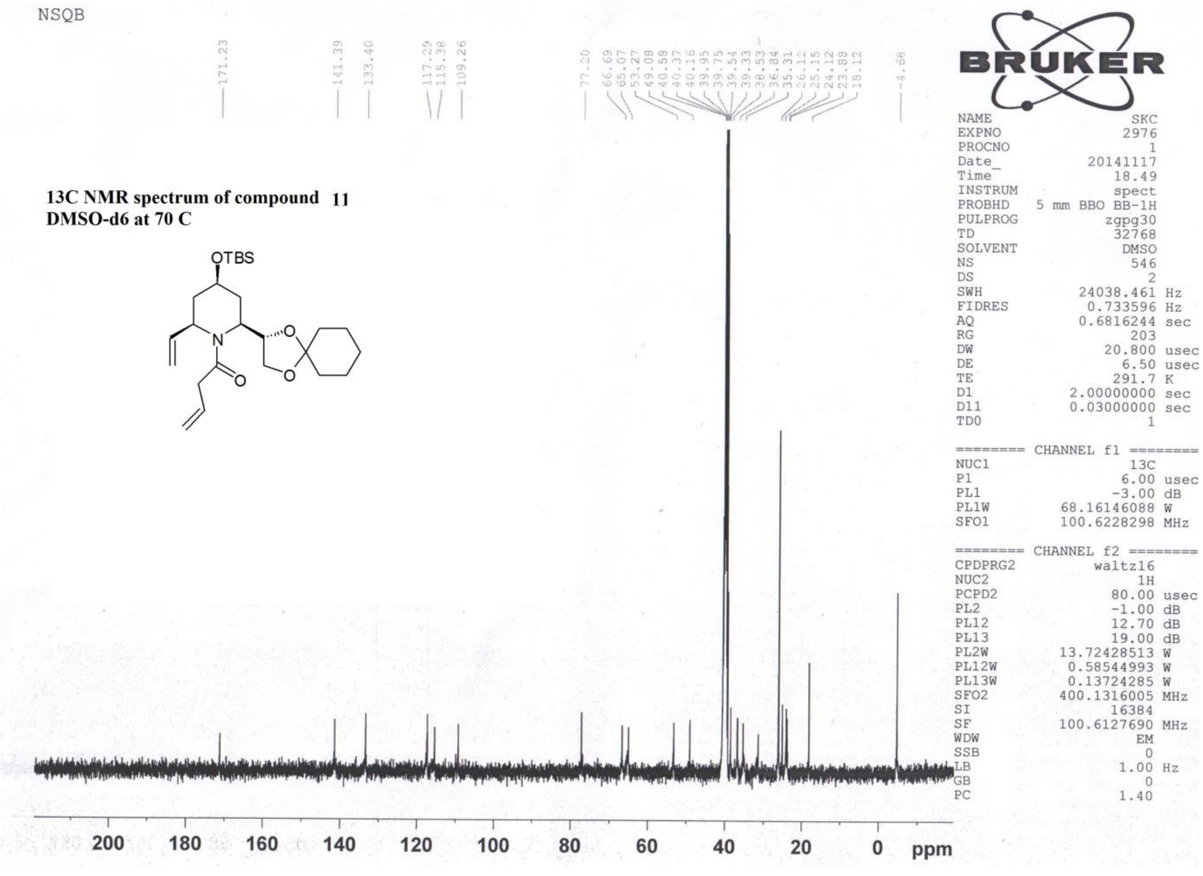
NSQB
13C NMR spectrum of compound 11
DMSO-d6 at 70 C
OTBS
N
O
O
O
BRUKER
NAME SKC
EXPNO 2976
PROCNO 1
Date_ 20141117
Time 18.49
INSTRUM spect
PROBHD 5 mm BBO BB-1H
PULPROG zgpg30
TD 32768
SOLVENT DMSO
NS 546
DS 2
SWH 24038.461 Hz
FIDRES 0.733596 Hz
AQ 0.6816244 sec
RG 203
DW 20.800 usec
DE 6.50 usec
TE 291.7 K
D1 2.00000000 sec
D11 0.03000000 sec
TD0 1
======== CHANNEL f1 ========
NUC1 13C
P1 6.00 usec
PL1 -3.00 dB
PL1W 68.16146088 W
SFO1 100.6228298 MHz
======== CHANNEL f2 ========
CPDPRG2 waltz16
NUC2 1H
PCPD2 80.00 usec
PL2 -1.00 dB
PL12 12.70 dB
PL13 19.00 dB
PL2W 13.72428513 W
PL12W 0.58544993 W
PL13W 0.13724285 W
SFO2 400.1316005 MHz
SI 16384
SF 100.6127690 MHz
WDW EM
SSB 0
LB 1.00 Hz
GB 0
PC 1.40
ppm

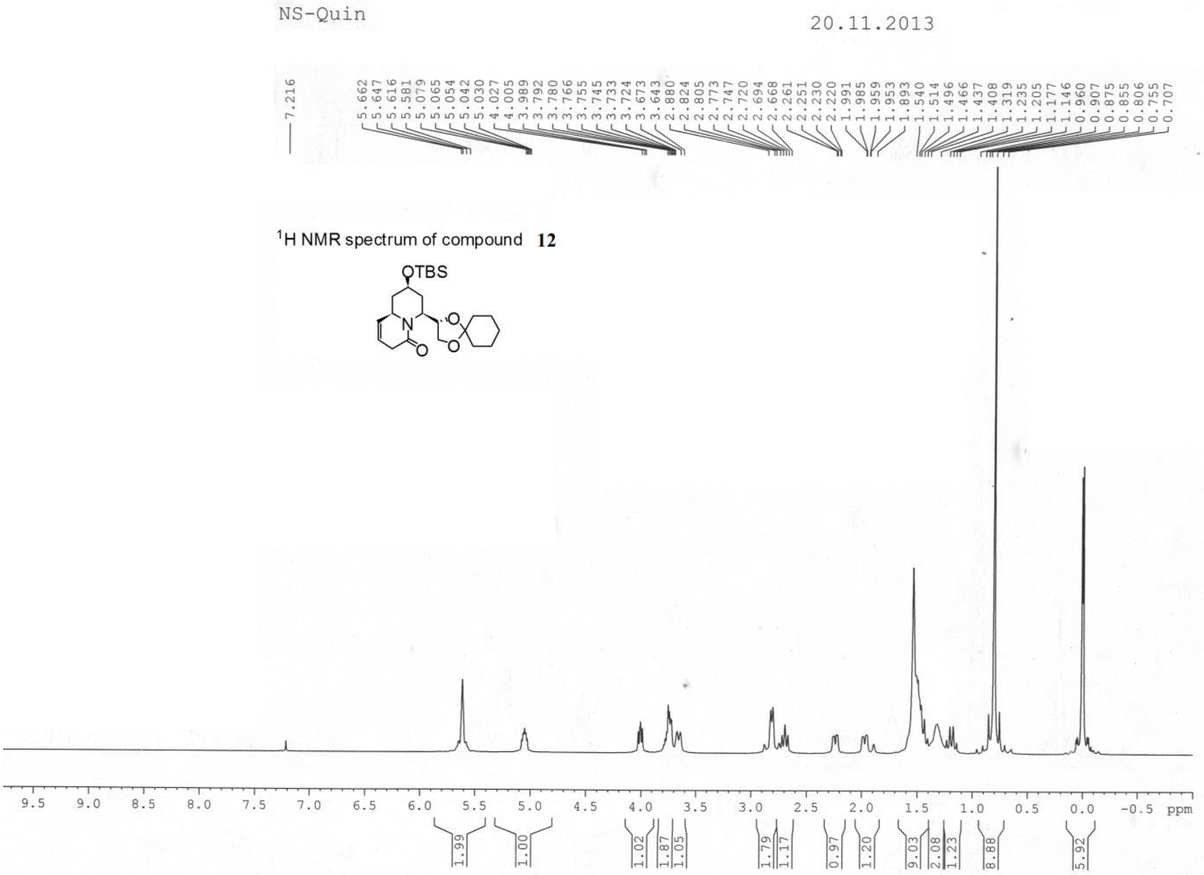
NS-Quin
20.11.2013
7.216
5.662 5.647 5.616 5.581 5.079 5.065 5.054 5.042 5.030 4.027 4.005 3.989 3.792 3.780 3.766 3.755 3.745 3.733 3.724 3.673 3.643 2.880 2.824 2.805 2.773 2.747 2.720 2.694 2.668 2.261 2.251 2.230 2.220 1.991 1.985 1.959 1.953 1.893 1.540 1.514 1.496 1.466 1.437 1.408 1.319 1.235 1.205 1.177 1.146 0.960 0.907 0.875 0.855 0.806 0.755 0.707
1H NMR spectrum of compound 12
OTBS
N
O
O
O
1.99 1.00 1.02 1.87 1.05 1.79 1.17 0.97 1.20 9.03 2.08 1.23 8.88 5.92
ppm

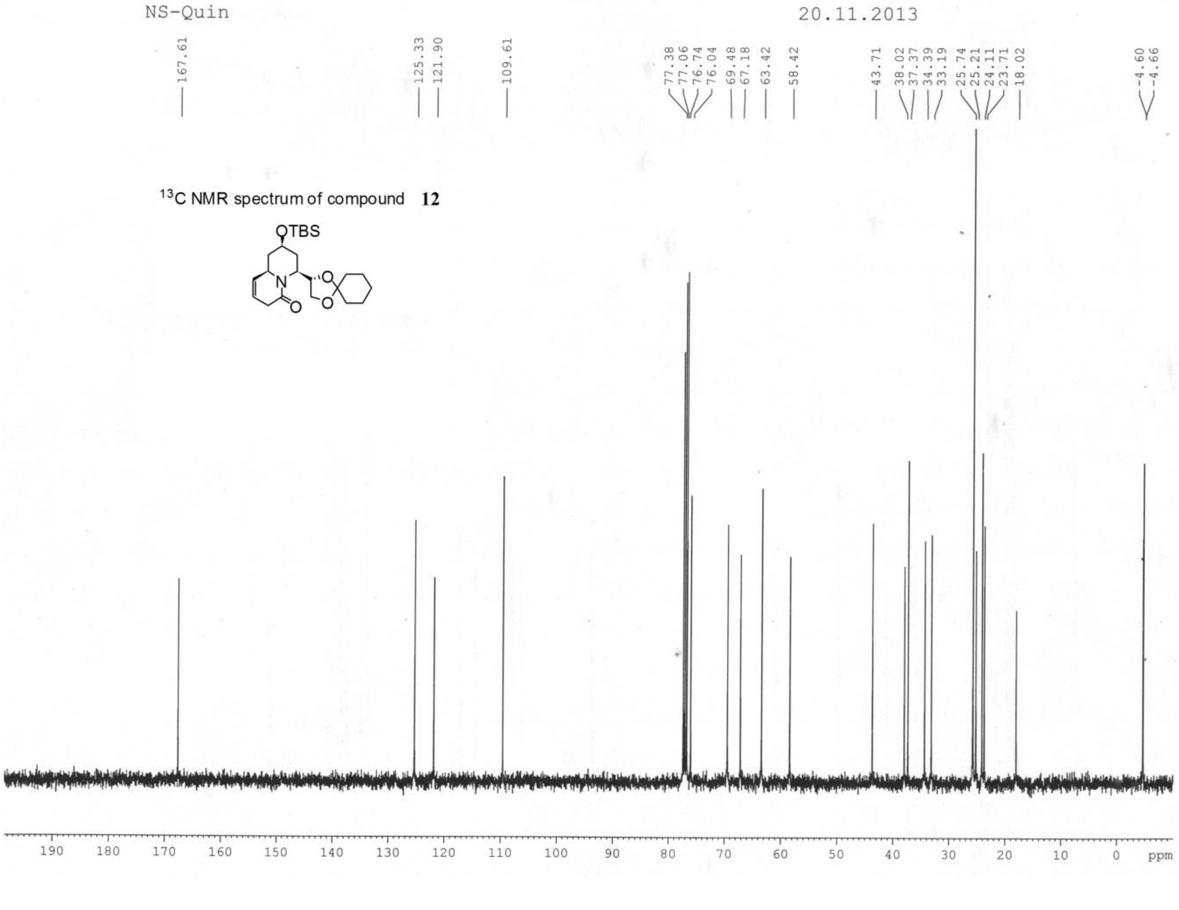
NS-Quin
20.11.2013
167.61
125.33 121.90
109.61
77.38 77.06 76.74 76.04 69.48 67.18 63.42 58.42
43.71 38.02 37.37 34.39 33.19 25.74 25.21 24.11 23.71 18.02
-4.60 -4.66
13C NMR spectrum of compound 12
OTBS
N
O
O
O
ppm

1H NMR spectrum of compound 13

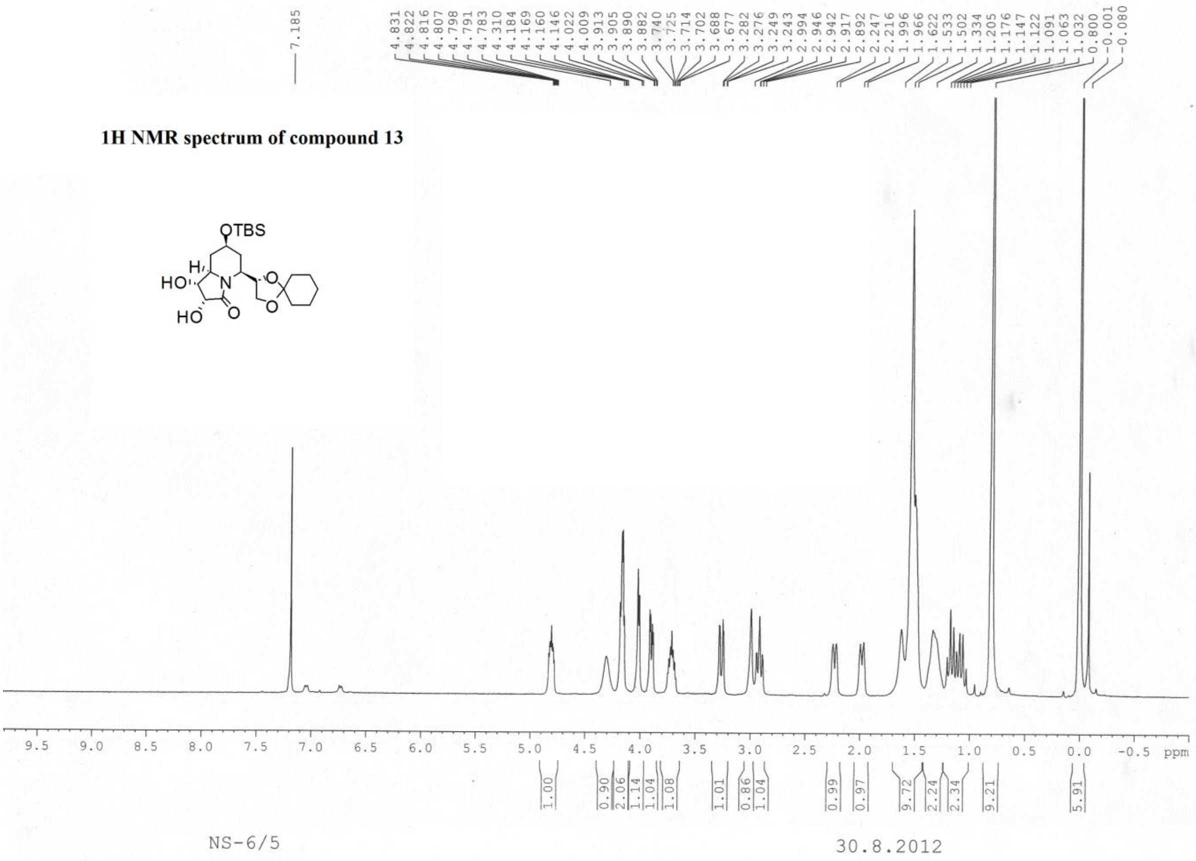

13C NMR spectrum of compound 13

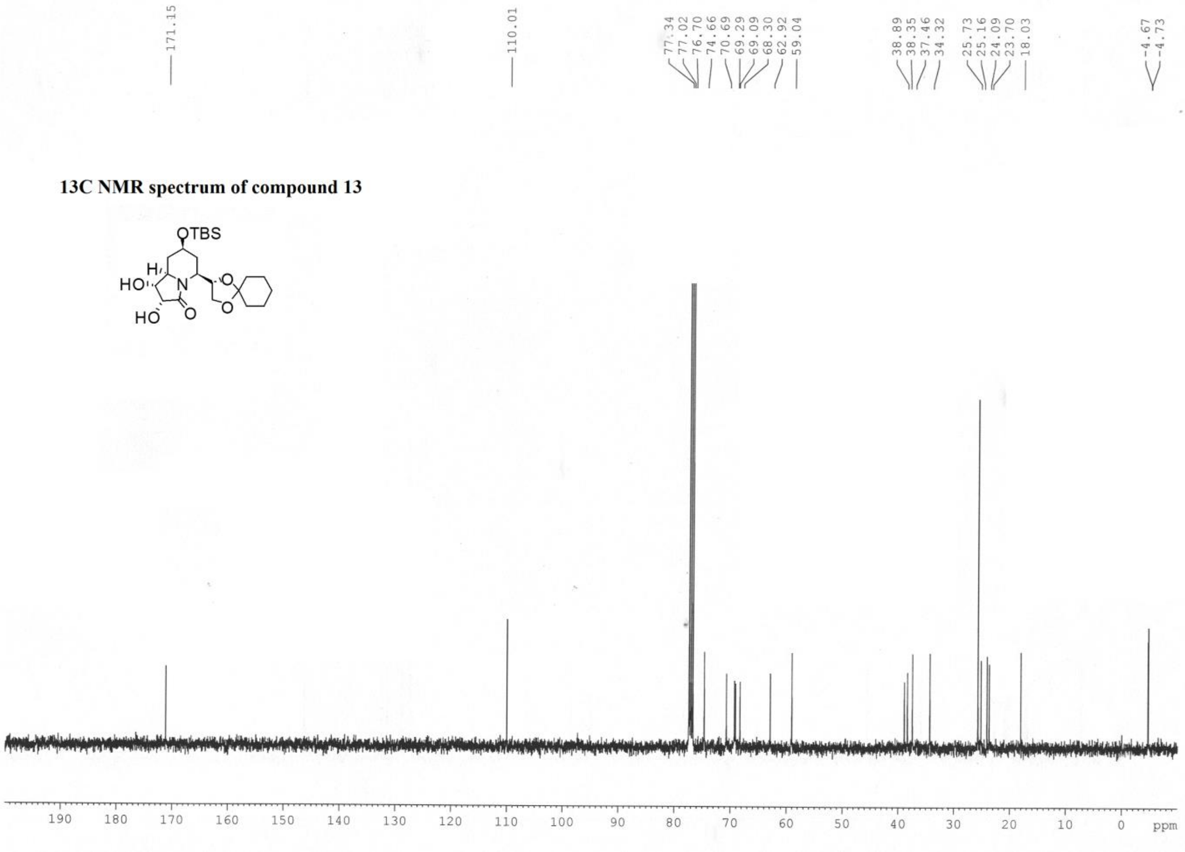

1H NMR spectrum of compound 14

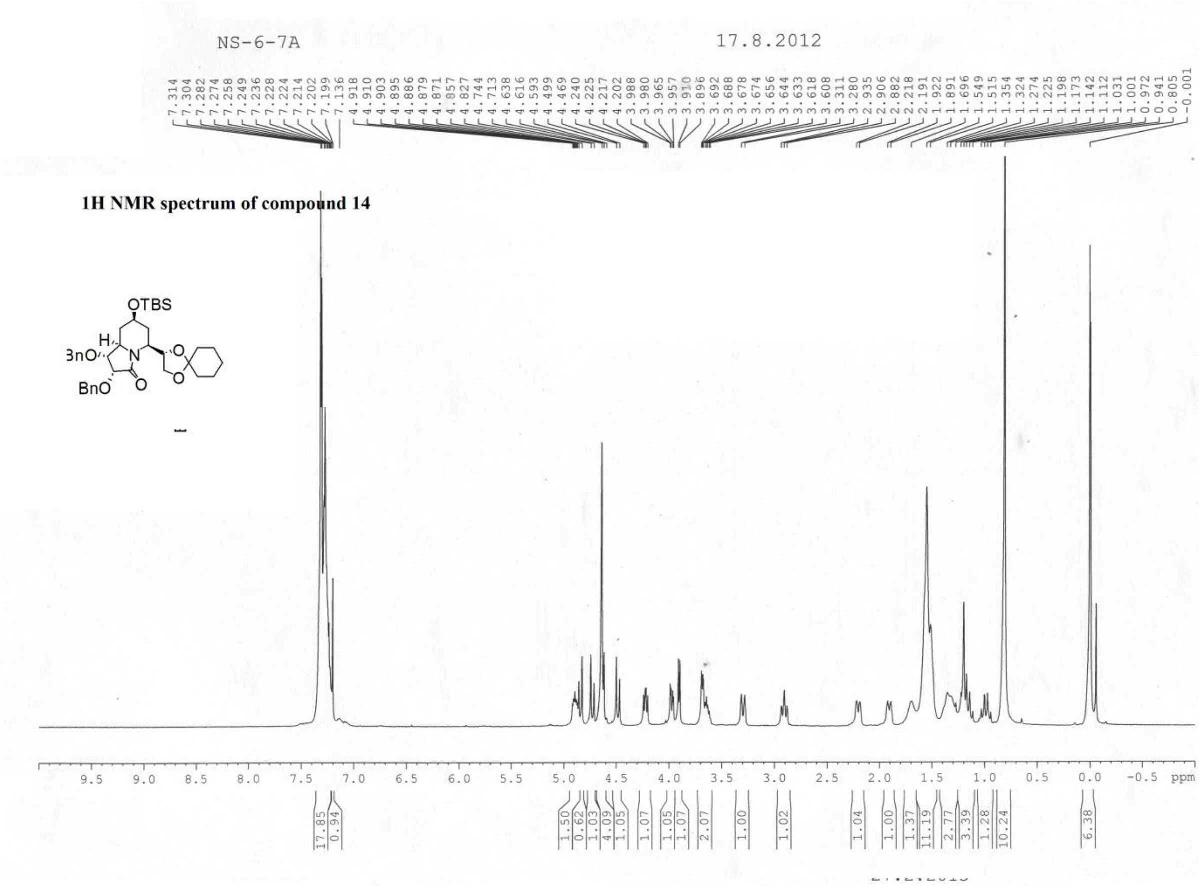

13C NMR spectrum of compound 14

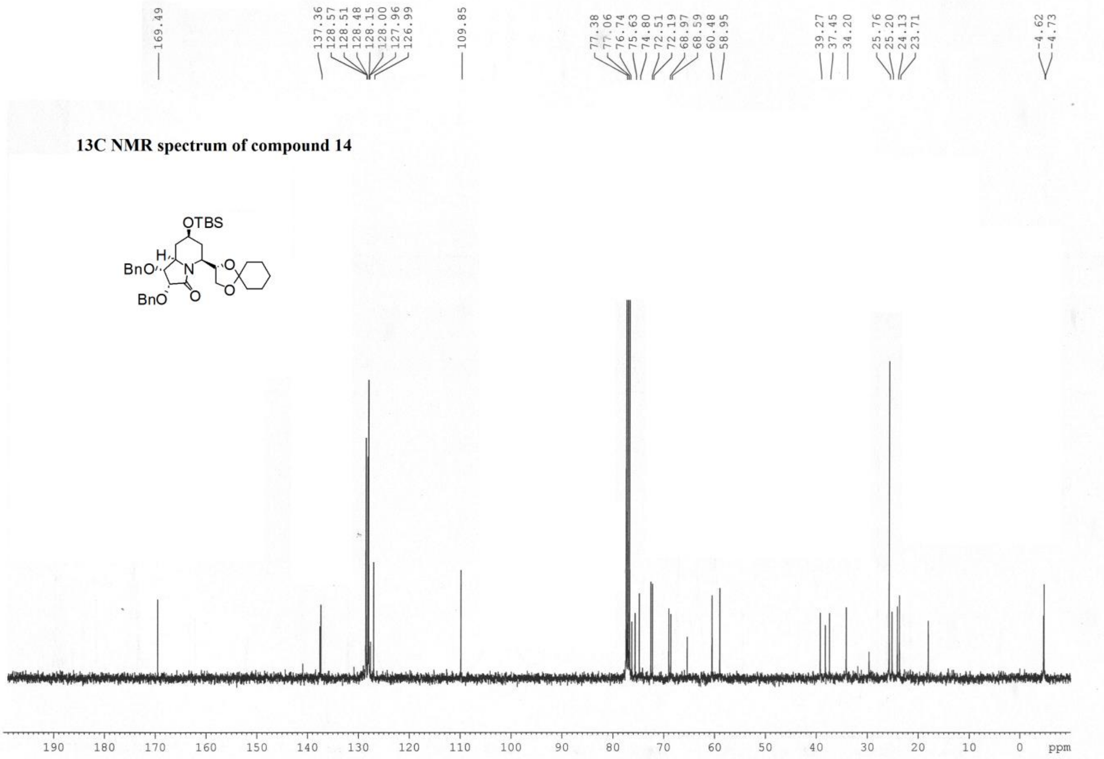

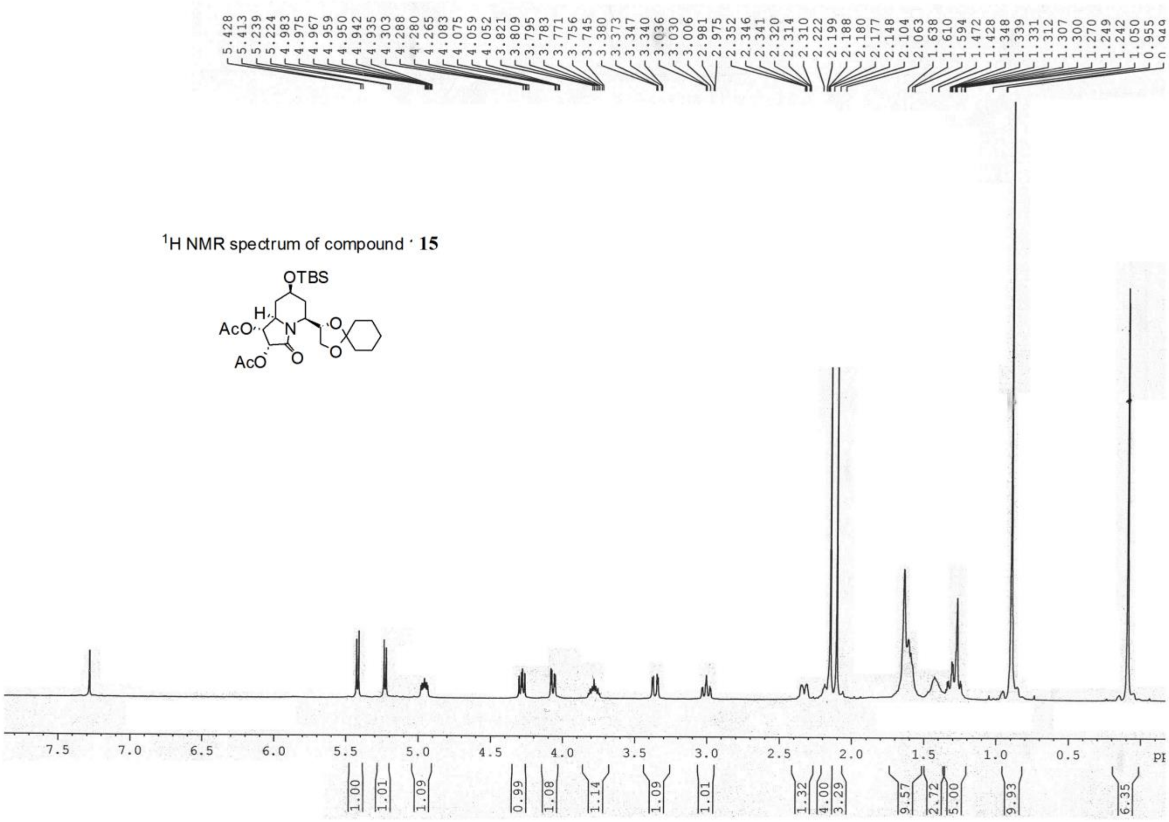

1H NMR spectrum of compound 15
OTBS
H
AcO
N
O
AcO
5.428 5.413 5.239 5.224 4.983 4.975 4.967 4.959 4.950 4.942 4.935 4.303 4.288 4.280 4.265 4.083 4.075 4.059 4.052 3.821 3.809 3.795 3.783 3.771 3.756 3.745 3.380 3.373 3.347 3.340 3.036 3.030 3.006 2.981 2.975 2.352 2.346 2.341 2.320 2.314 2.310 2.222 2.199 2.188 2.180 2.177 2.148 2.104 2.063 1.638 1.610 1.594 1.472 1.428 1.348 1.339 1.331 1.312 1.307 1.300 1.270 1.249 1.242 1.050 0.955
7.5 7.0 6.5 6.0 5.5 5.0 4.5 4.0 3.5 3.0 2.5 2.0 1.5 1.0 0.5
1.00 1.01 1.09 0.99 1.08 1.14 1.09 1.01 1.32 4.00 3.29 9.57 2.72 5.00 9.93 6.35

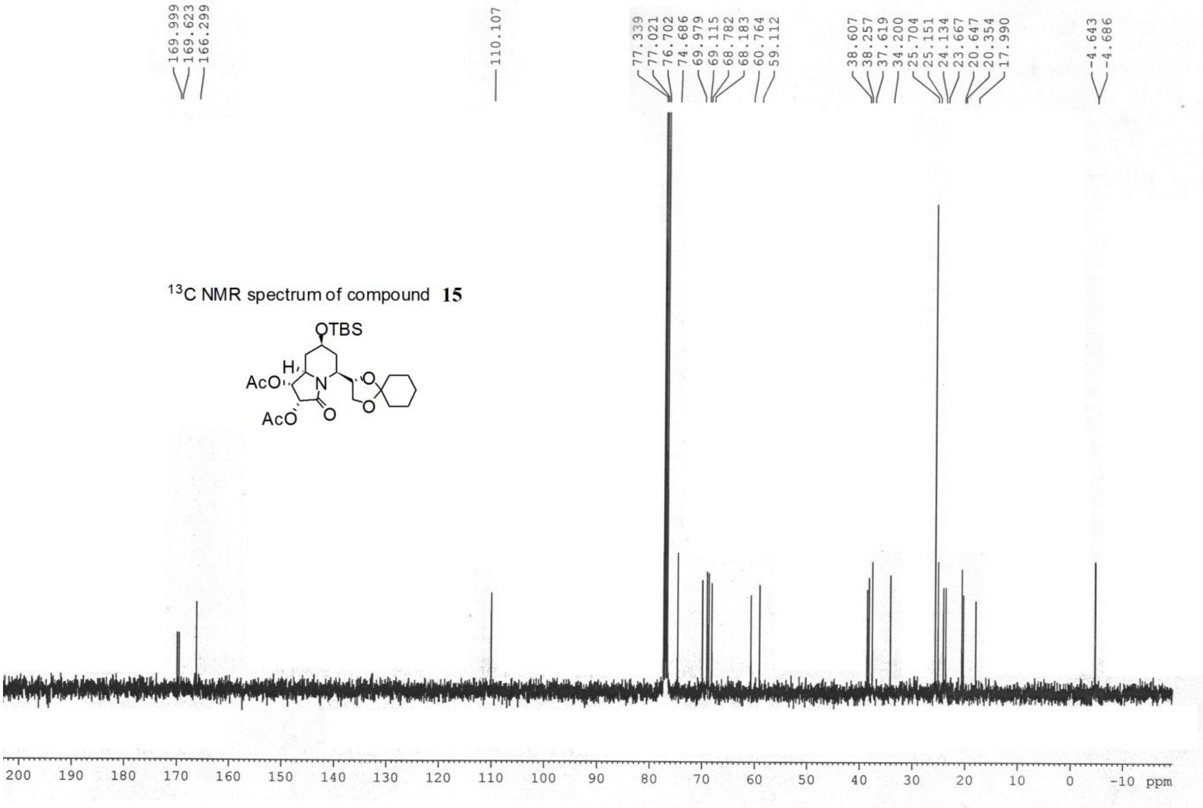

13C NMR spectrum of compound 15
OTBS
H
AcO
N
O
AcO
169.999 169.623 166.299
110.107
77.339 77.021 76.702 74.686 69.979 69.115 68.782 68.183 60.764 59.112
38.607 38.257 37.619 34.200 25.704 25.151 24.134 23.667 20.647 20.354 17.990
-4.643 -4.686
200 190 180 170 160 150 140 130 120 110 100 90 80 70 60 50 40 30 20 10 0 -10 ppm

1H-1H COSY spectrum of compound 15

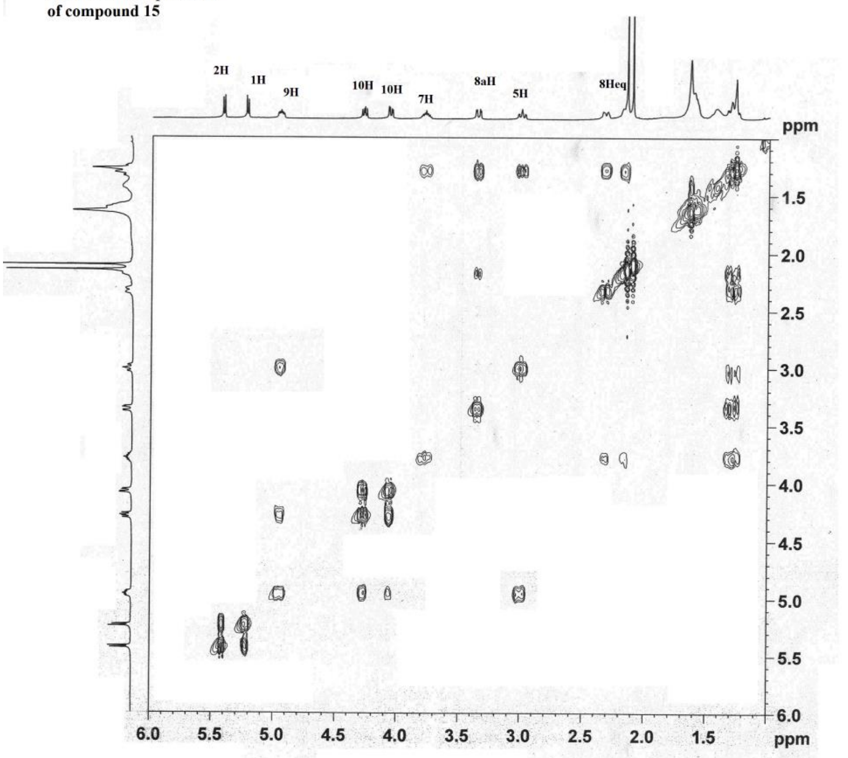

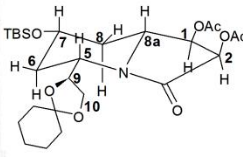

COSY partnar
1H > 2H
5H > 6Hax, 6Heq, 9H
7H > 6Hax, 6Heq, 8Hax, 8Heq
8aH > 8Hax, 8Heq

HSQC spectrum of compound 15

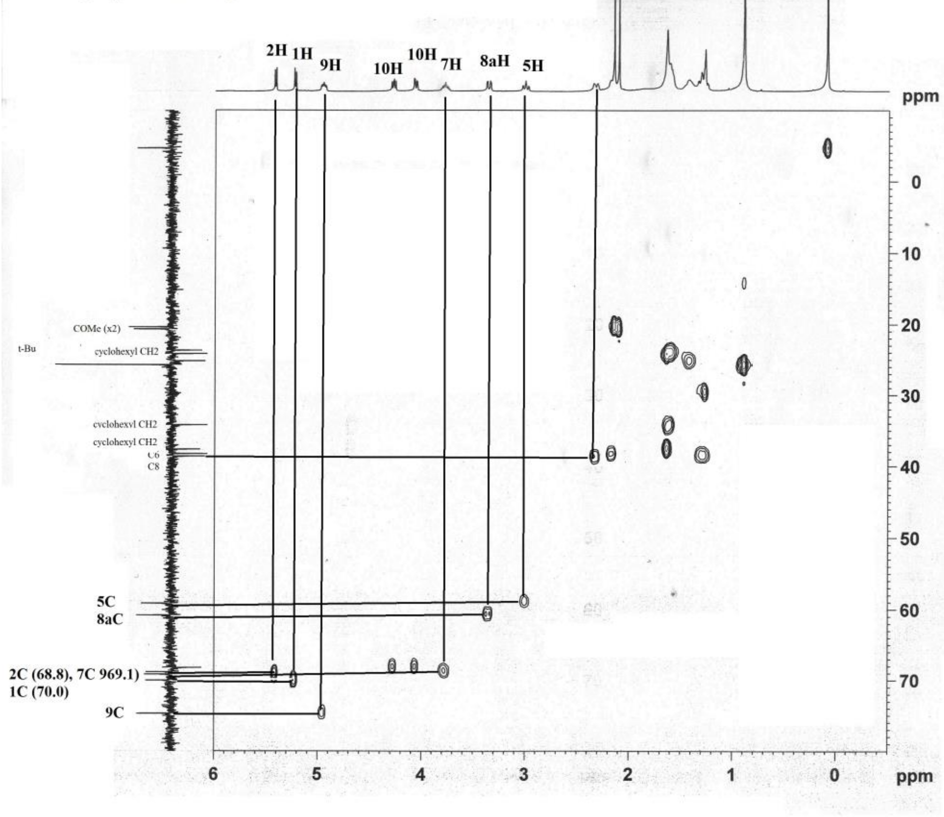

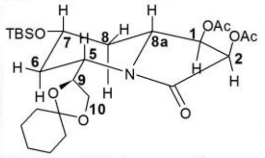

NOESY spectrum of compound 15

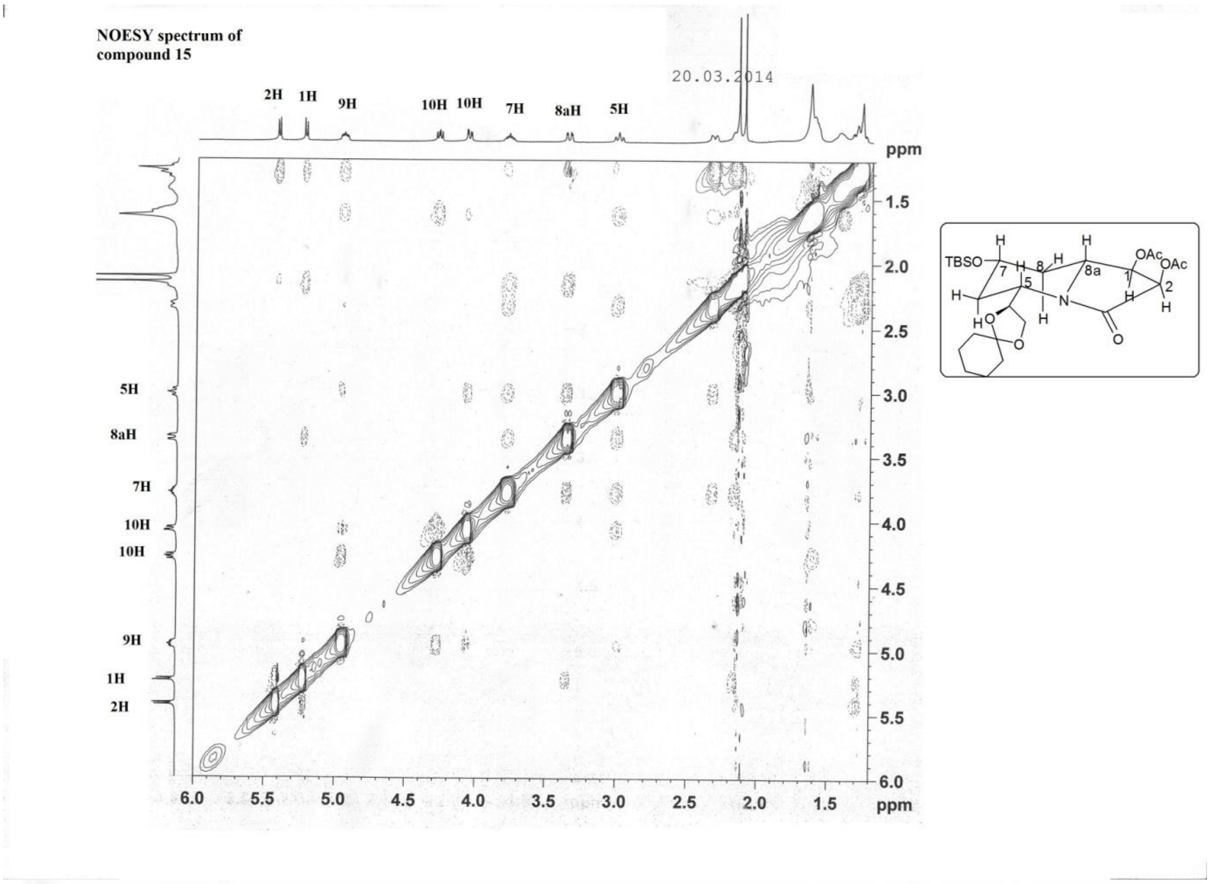

1H NMR spectrum of compound 16

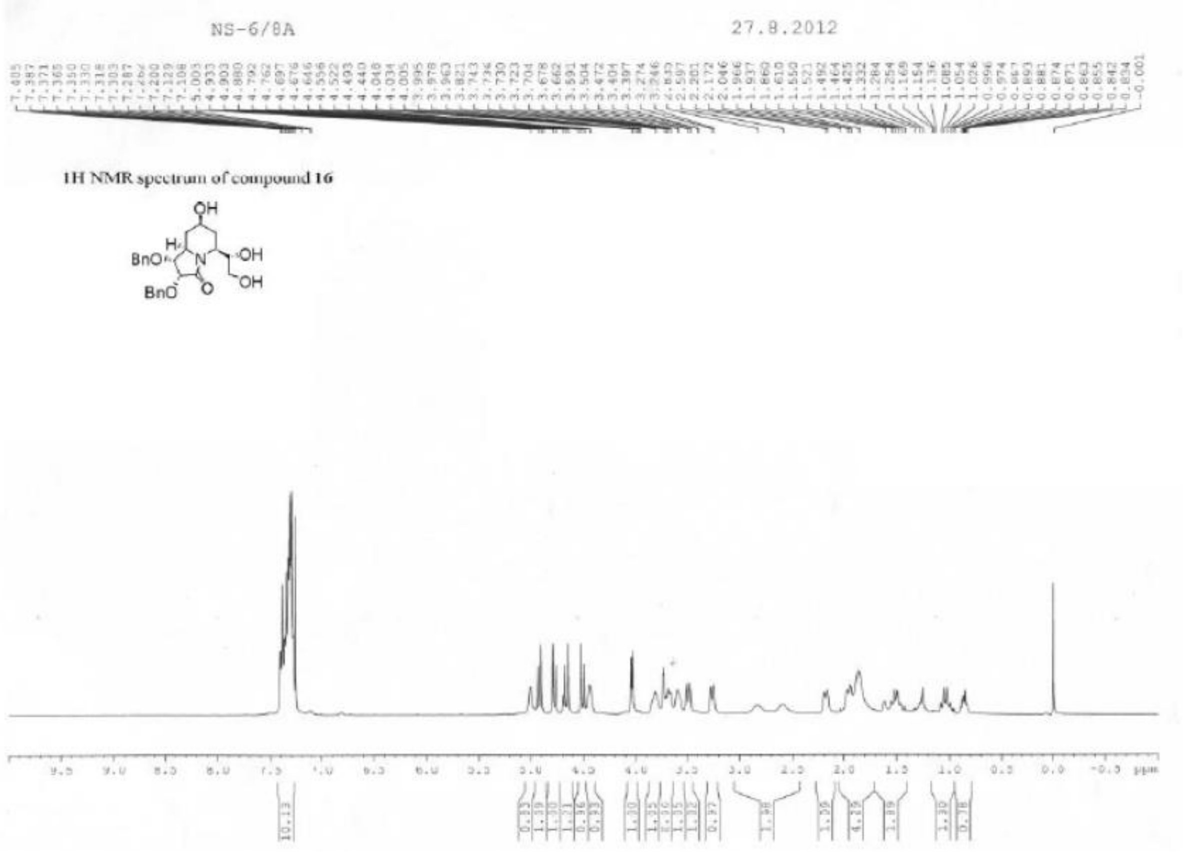

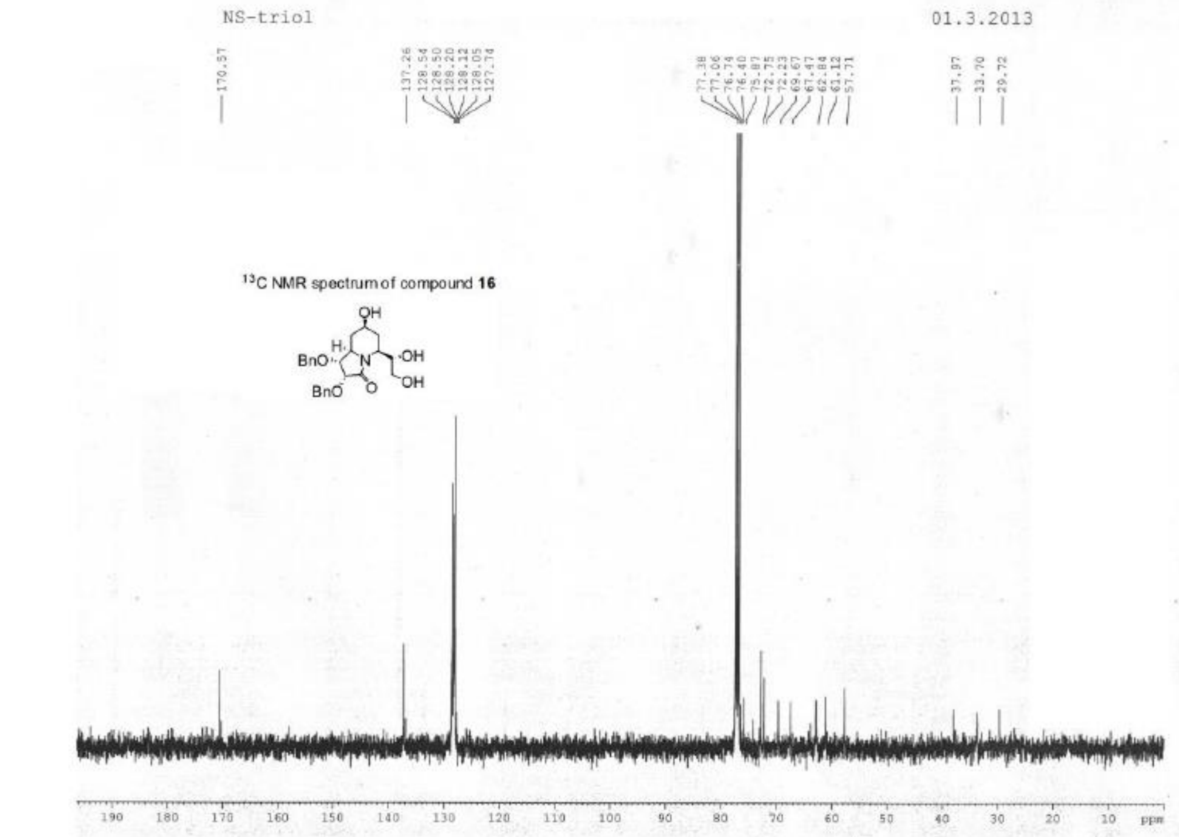
NS-triol
01.3.2013
170.57
137.26
128.54
128.50
128.20
128.12
128.05
127.74
77.38
77.06
76.74
76.40
75.87
72.75
72.23
69.67
67.47
62.84
61.12
57.71
37.97
33.70
29.72
^{13}C NMR spectrum of compound **16**
OH
H
BnO
N
OH
OH
BnO
O
ppm

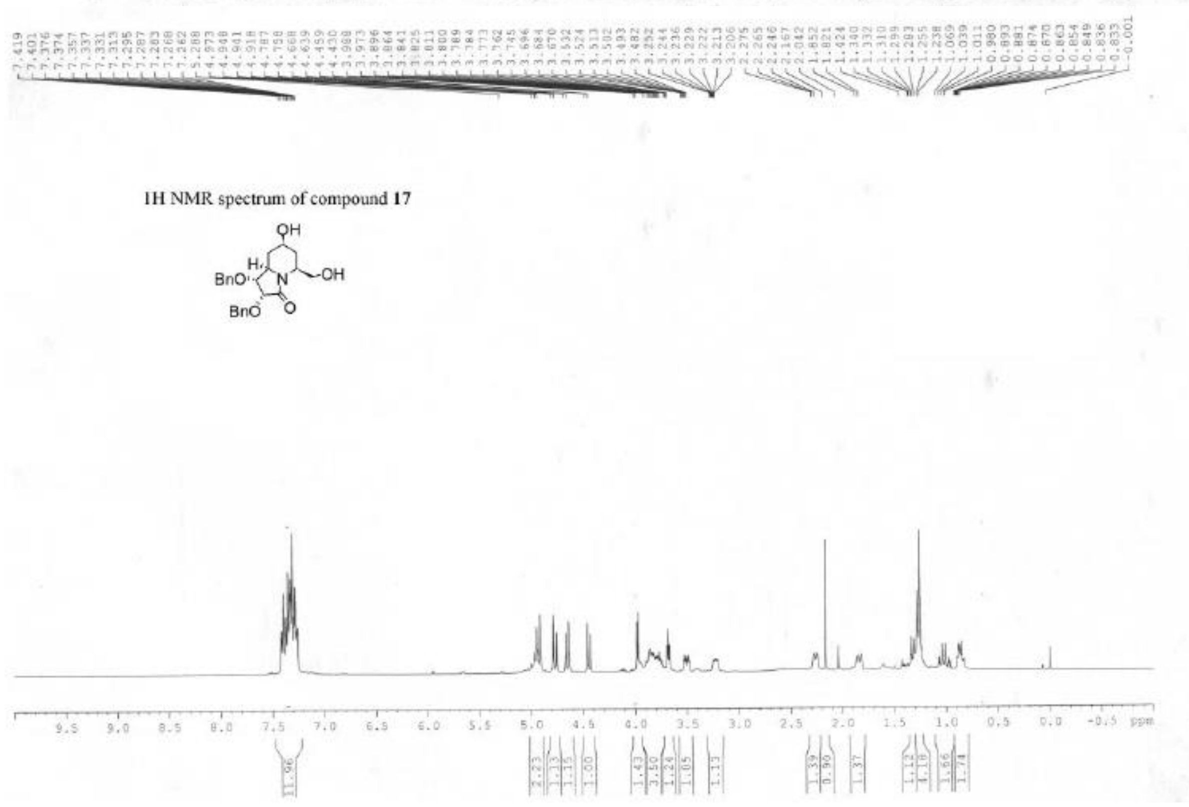
1H NMR spectrum of compound **17**
OH
H
BnO
N
OH
BnO
O
ppm

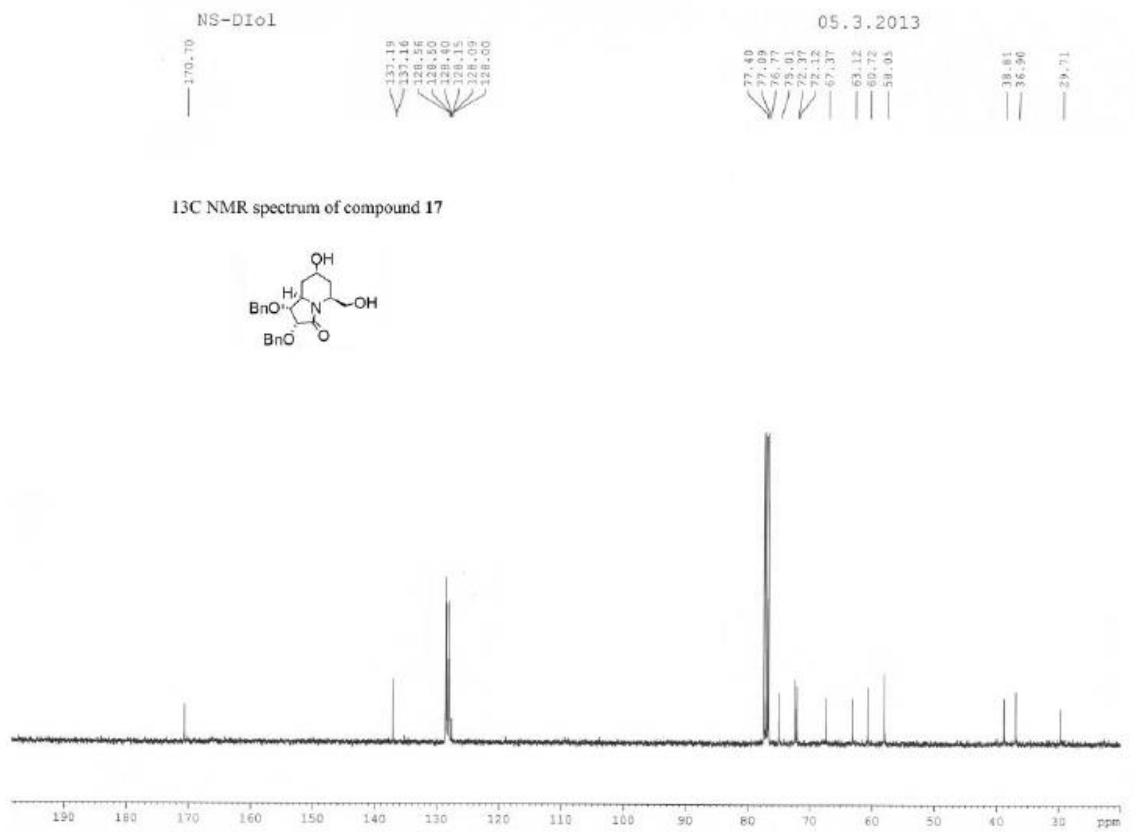

13C NMR spectrum of compound **17**

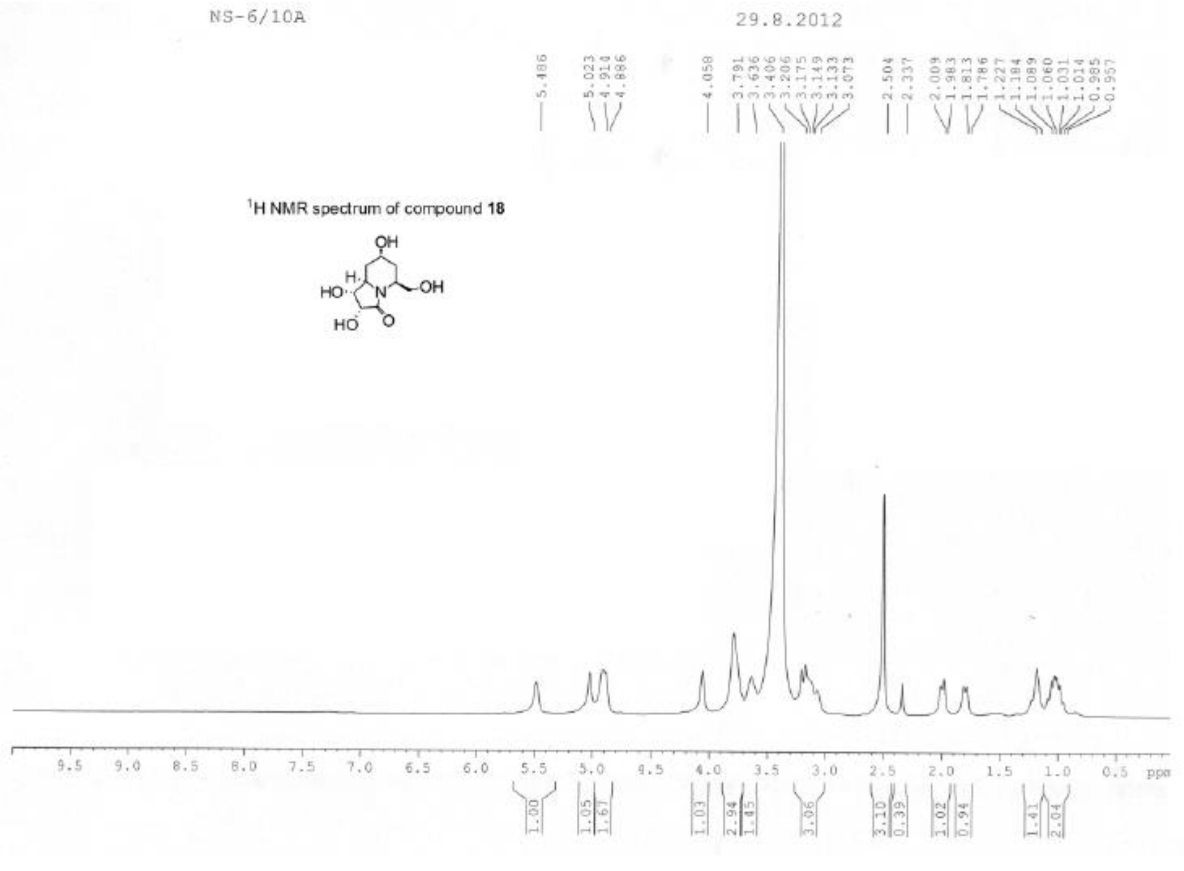

^{1}H NMR spectrum of compound **18**

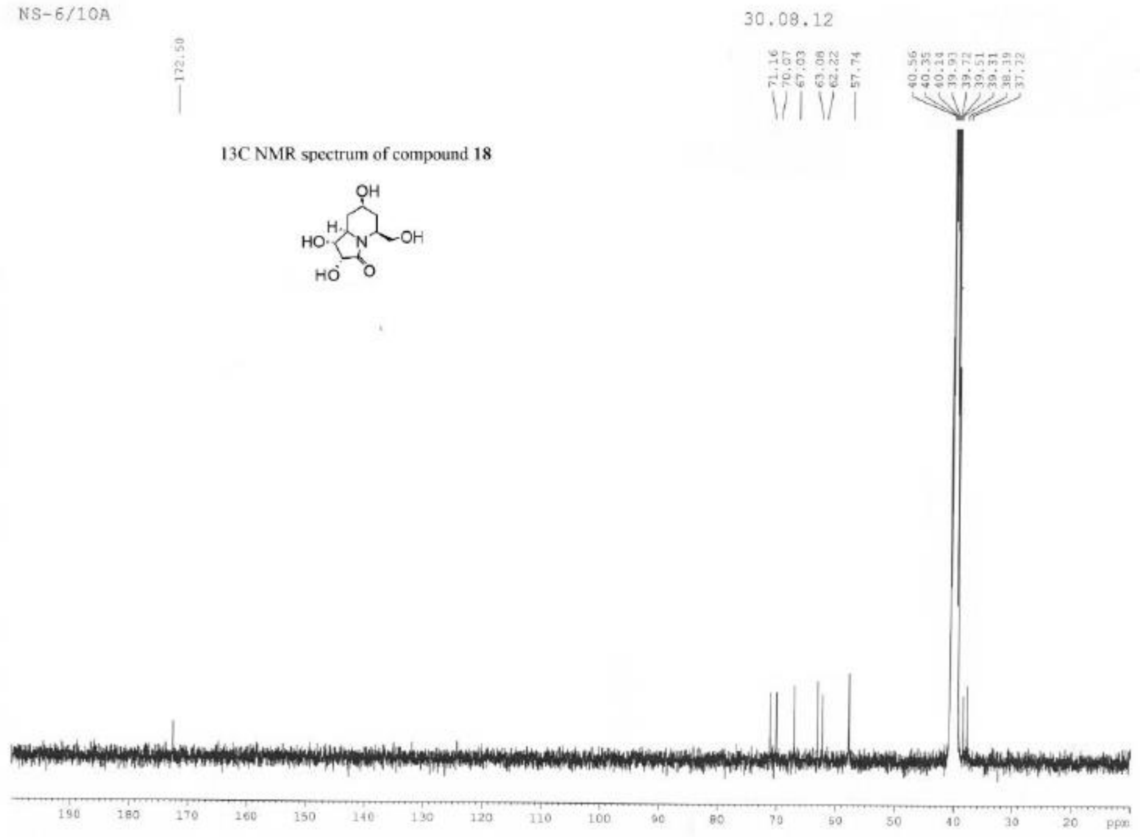

NS-6/10A
30.08.12
172.50
71.16
70.07
67.03
63.08
62.22
57.74
40.56
40.35
40.14
39.93
39.72
39.51
39.31
38.39
37.72
13C NMR spectrum of compound 18
OH
H
HO
N
OH
HO
O
190
180
170
160
150
140
130
120
110
100
90
80
70
60
50
40
30
20
ppm

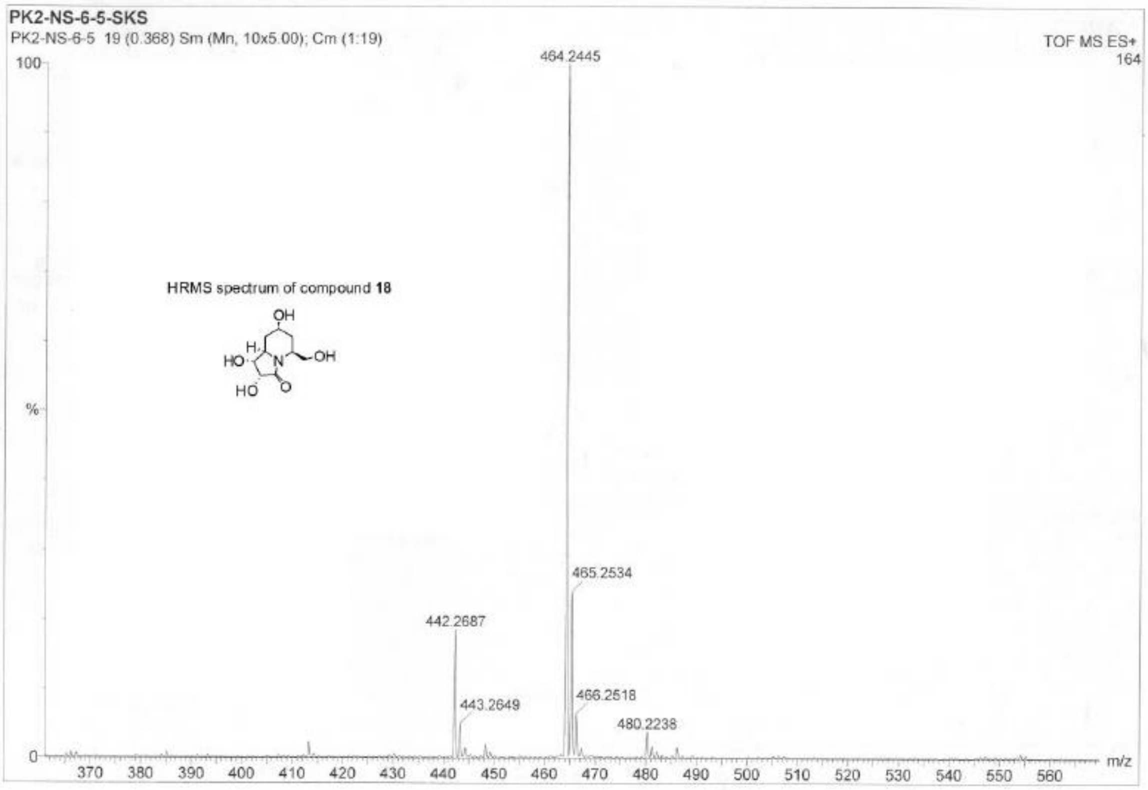

PK2-NS-6-5-SKS
PK2-NS-6-5 19 (0.368) Sm (Mn, 10x5.00); Cm (1:19)
TOF MS ES+
164
100
%
0
464.2445
465.2534
442.2687
443.2649
466.2518
480.2238
HRMS spectrum of compound 18
OH
H
HO
N
OH
HO
O
370
380
390
400
410
420
430
440
450
460
470
480
490
500
510
520
530
540
550
560
m/z

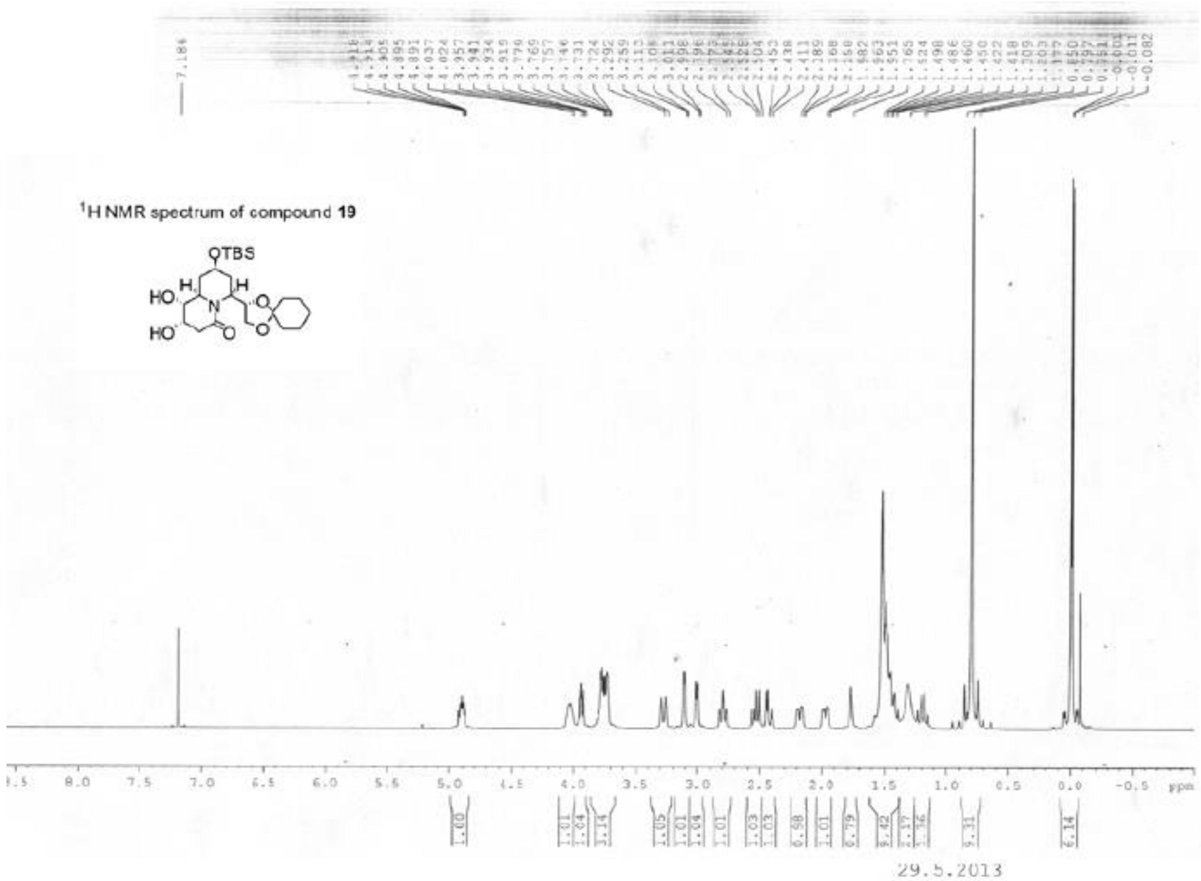

^{1}H NMR spectrum of compound **19**
OTBS
HO
HO
N
O
ppm
29.5.2013

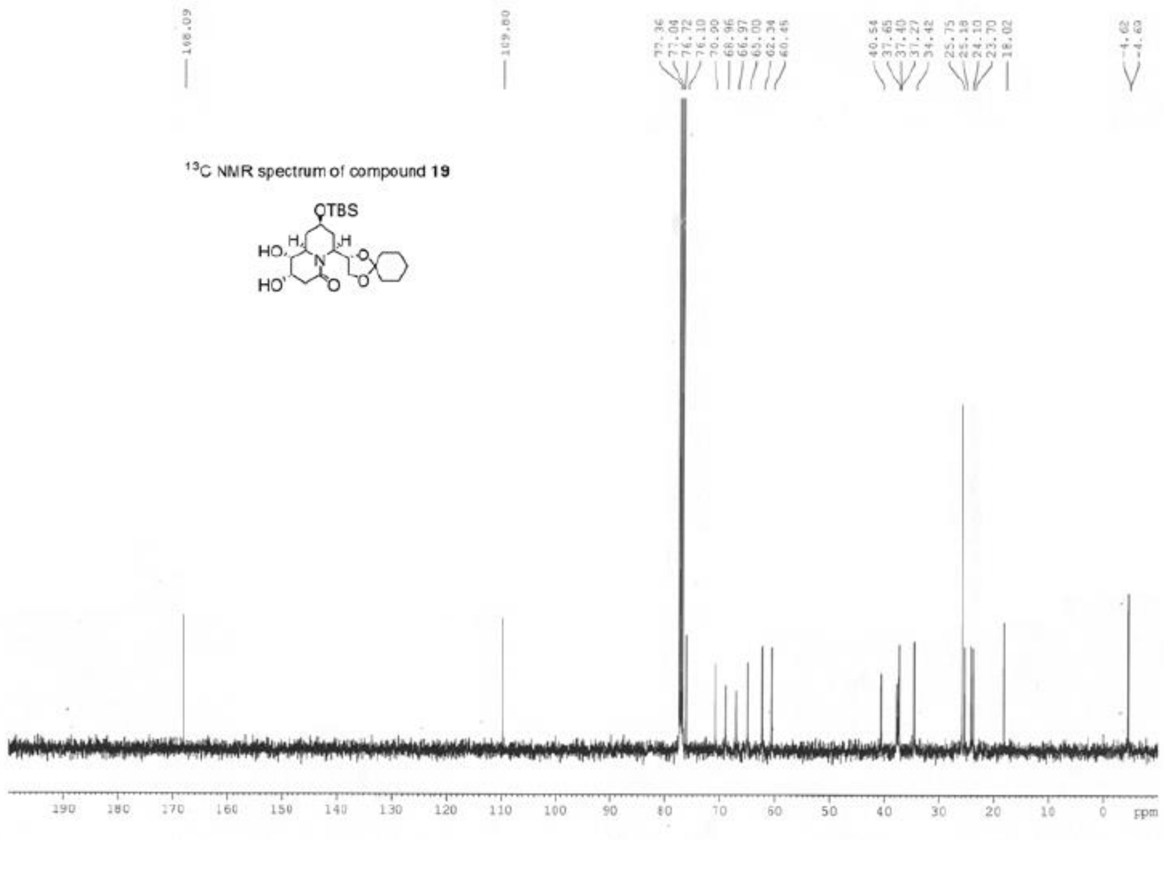

^{13}C NMR spectrum of compound **19**
OTBS
HO
HO
N
O
ppm

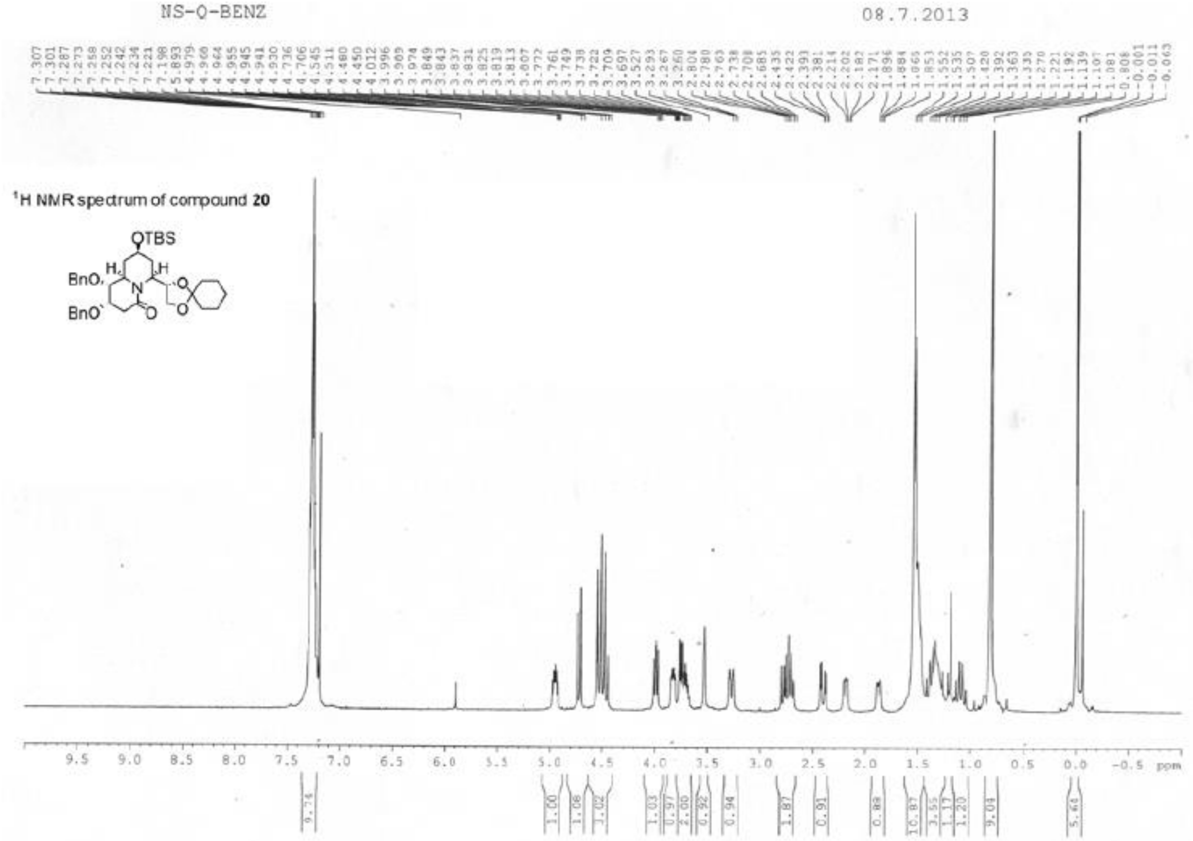
NS-Q-BENZ
08.7.2013
¹H NMR spectrum of compound 20
OTBS
BnO
BnO
ppm

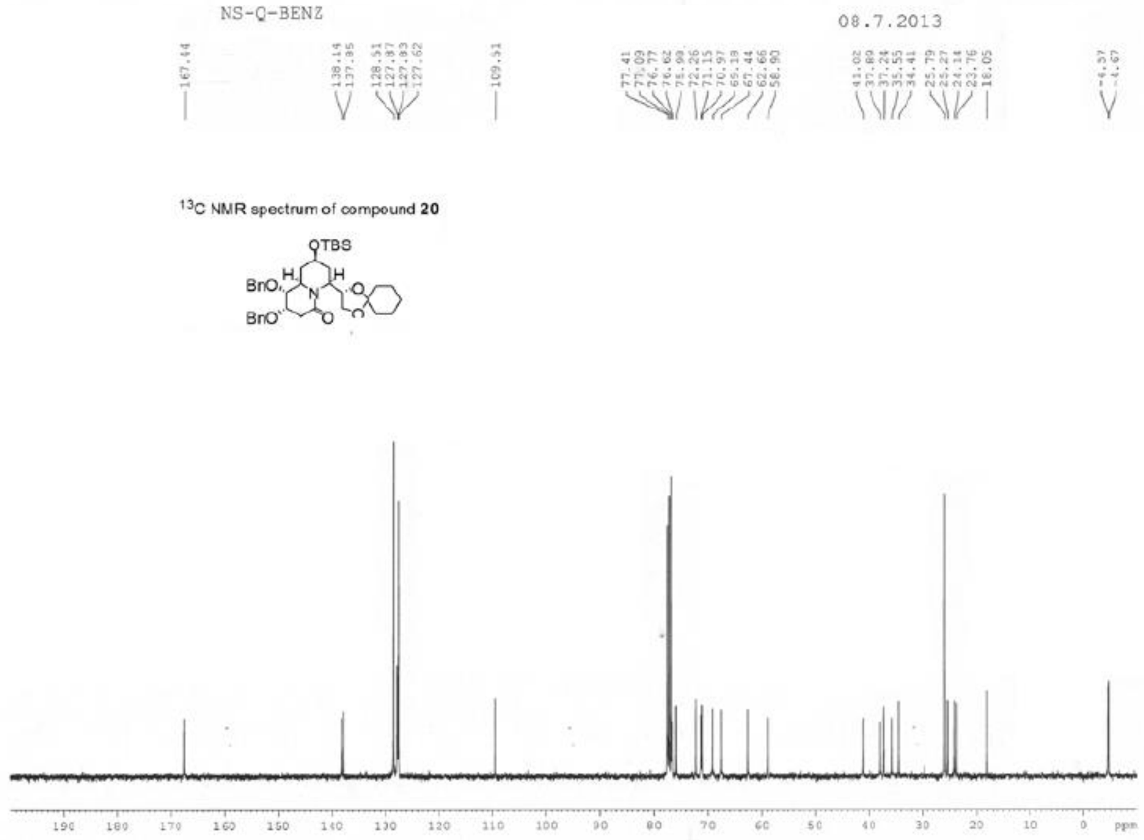
NS-Q-BENZ
08.7.2013
¹³C NMR spectrum of compound 20
OTBS
BnO
BnO
ppm

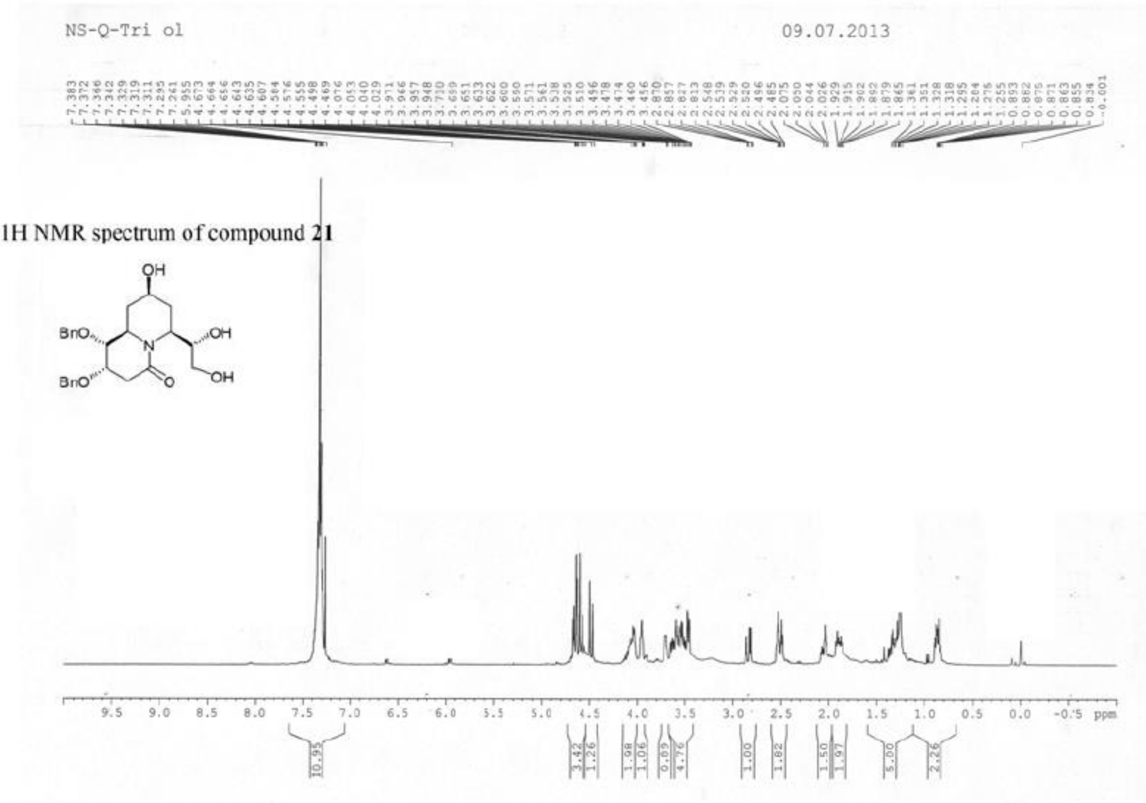

1H NMR spectrum of compound 21

13C NMR spectrum of compound 21

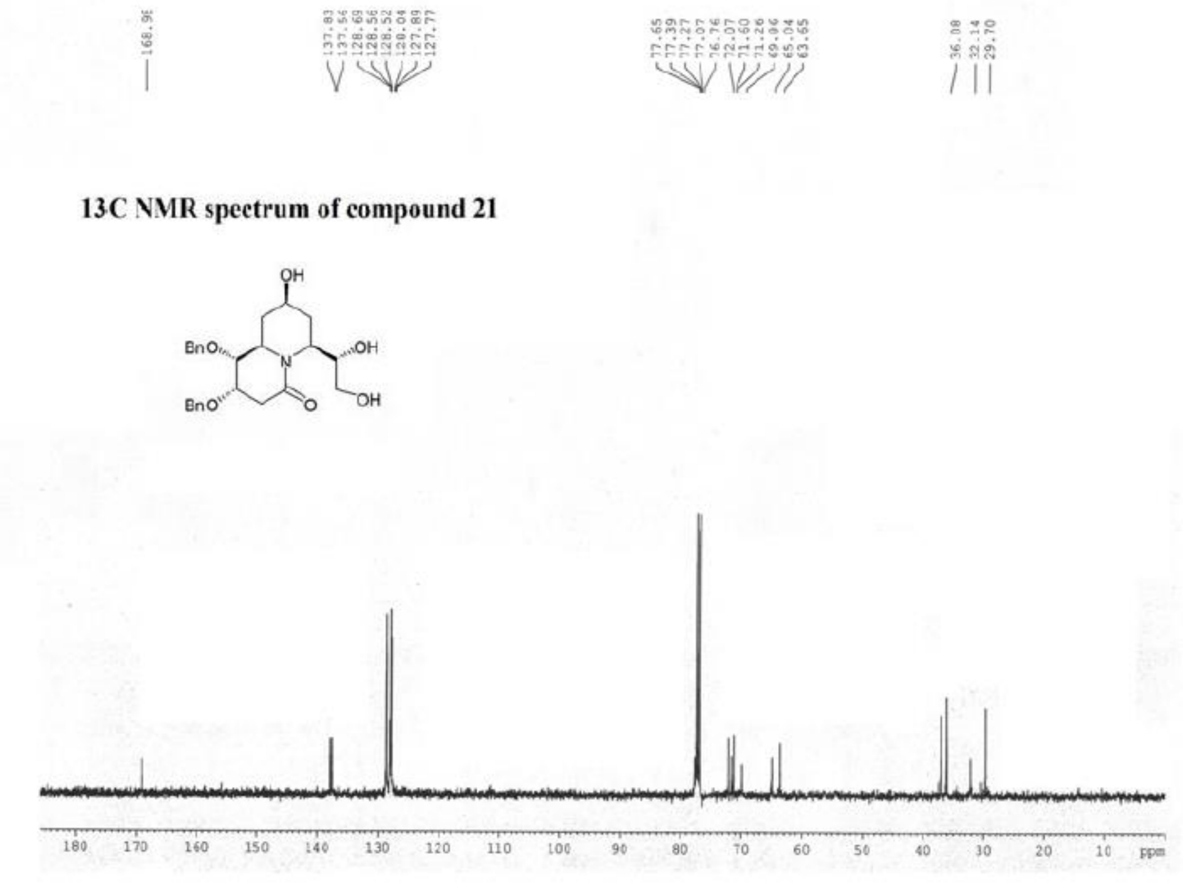

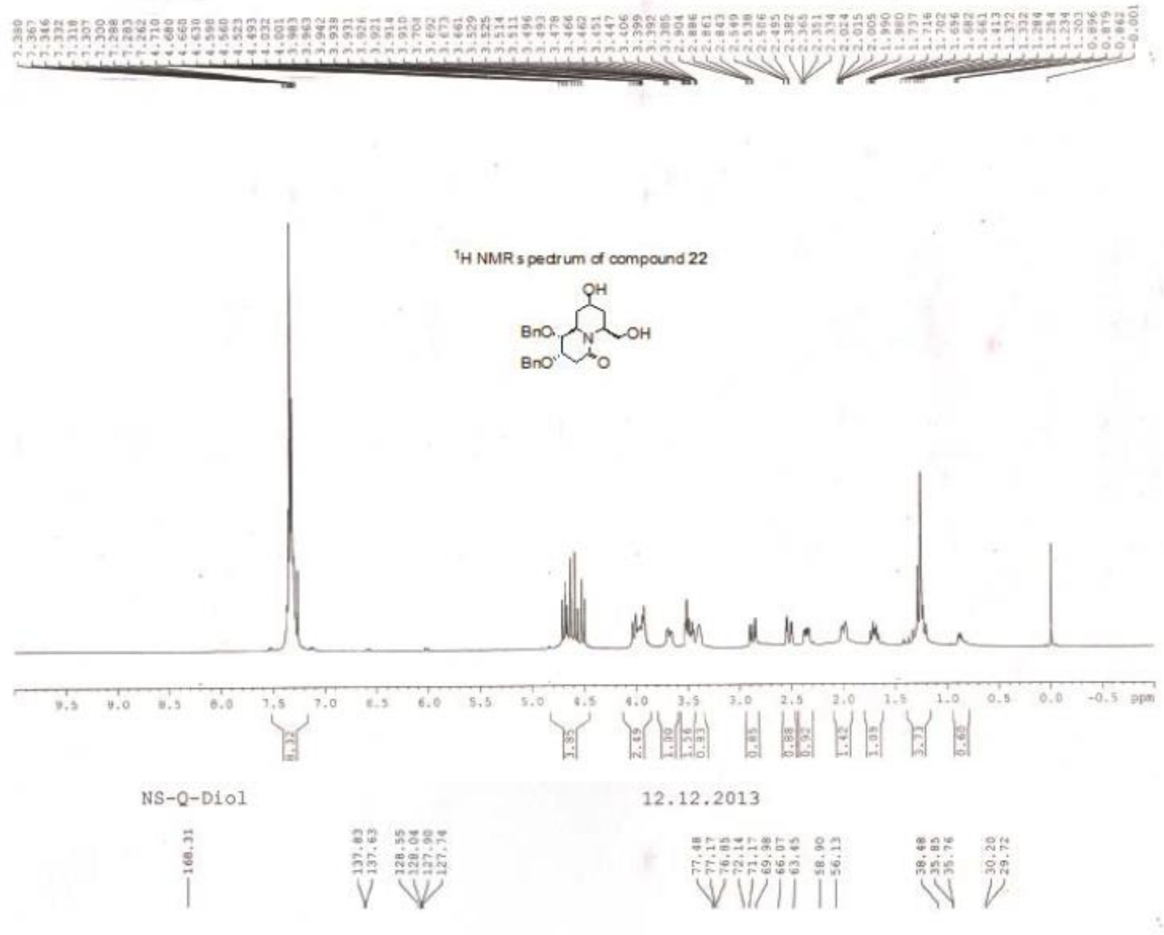

1H NMR spectrum of compound 22
NS-Q-Diol
12.12.2013

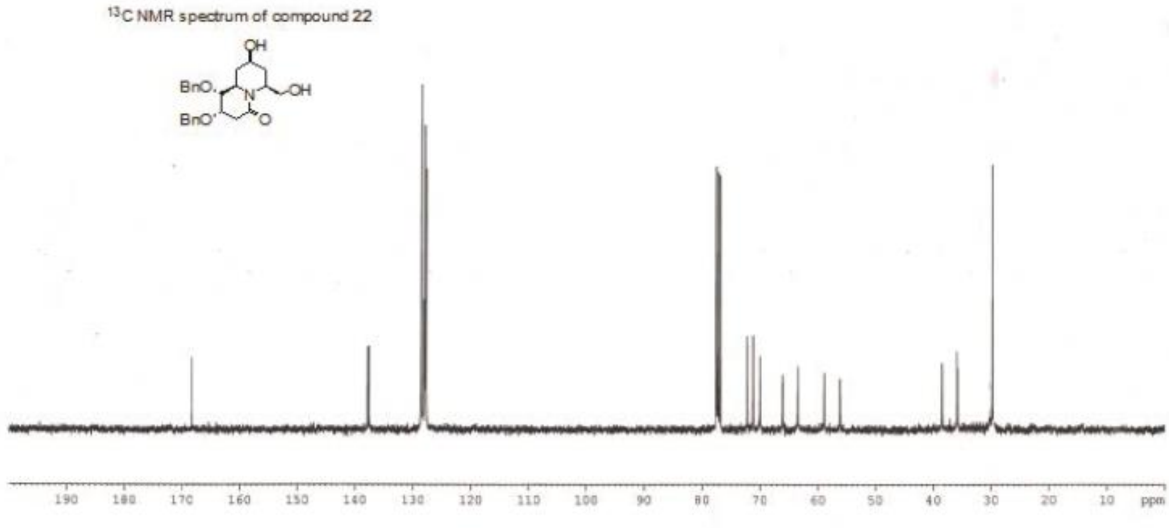

13C NMR spectrum of compound 22

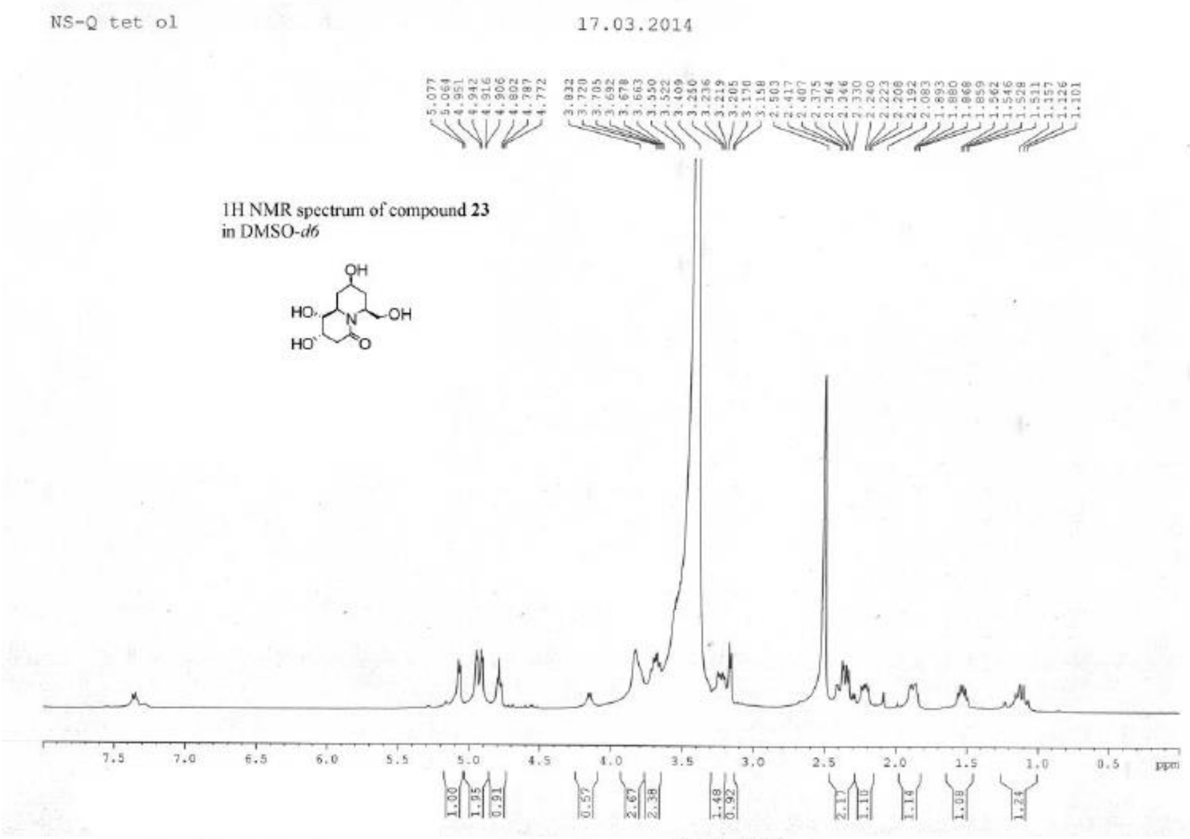

1H NMR spectrum of compound **23** in DMSO-*d6*

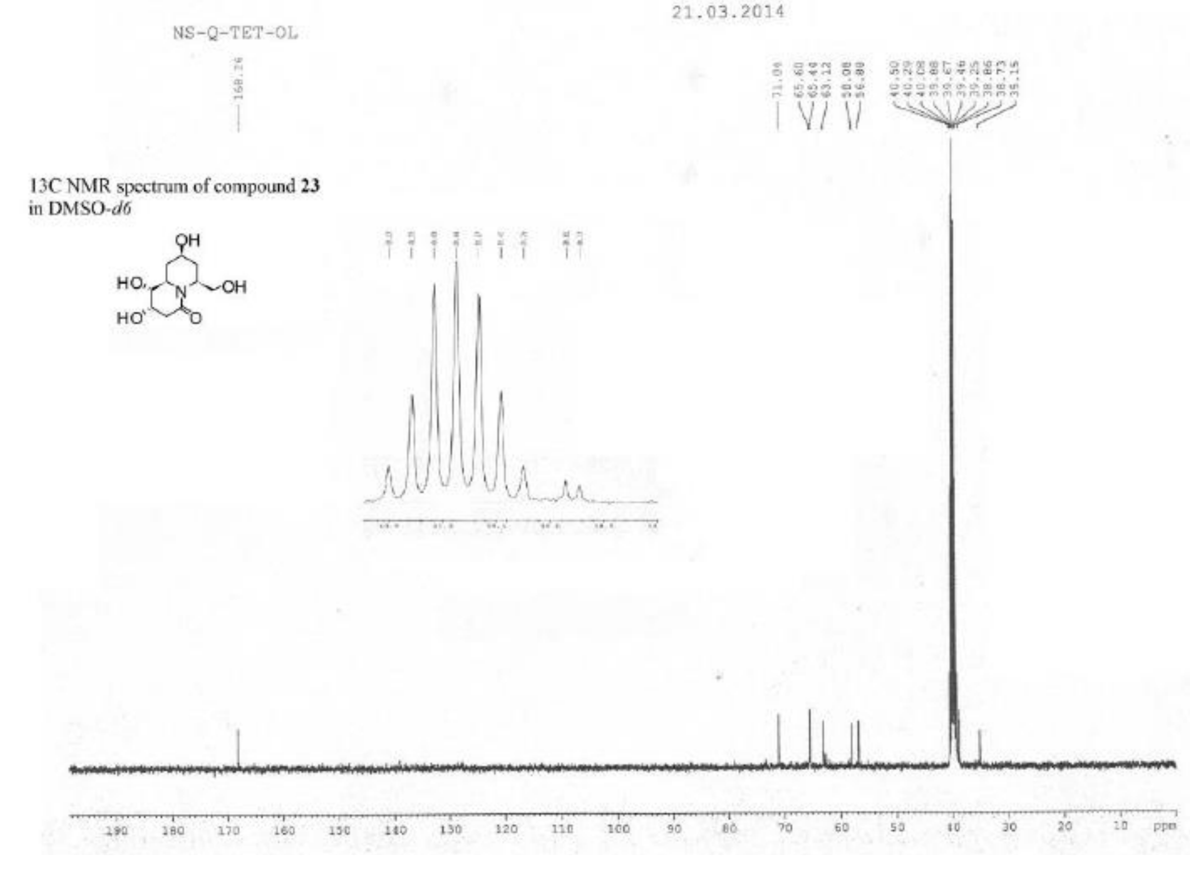

13C NMR spectrum of compound **23** in DMSO-*d6*

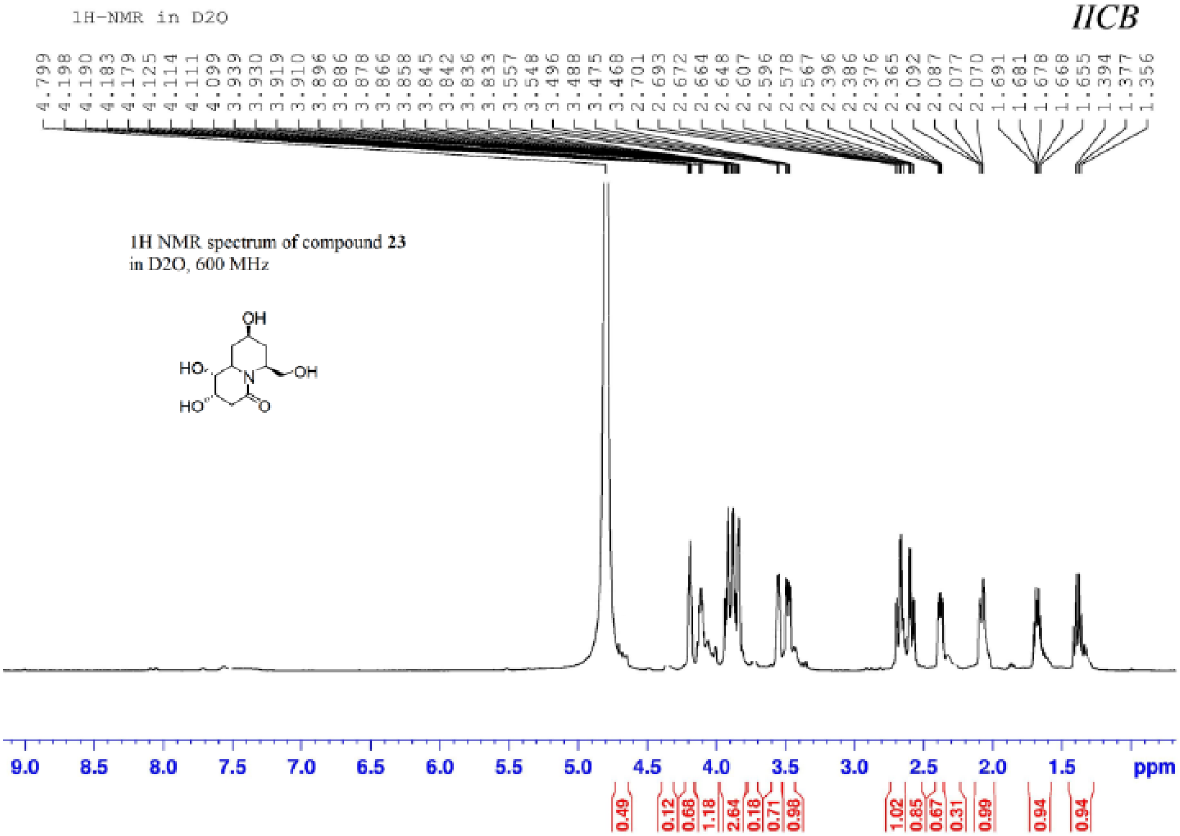

1H-NMR in D2O
IICB
1H NMR spectrum of compound 23
in D2O, 600 MHz
OH
HO
N
OH
HO
O
ppm

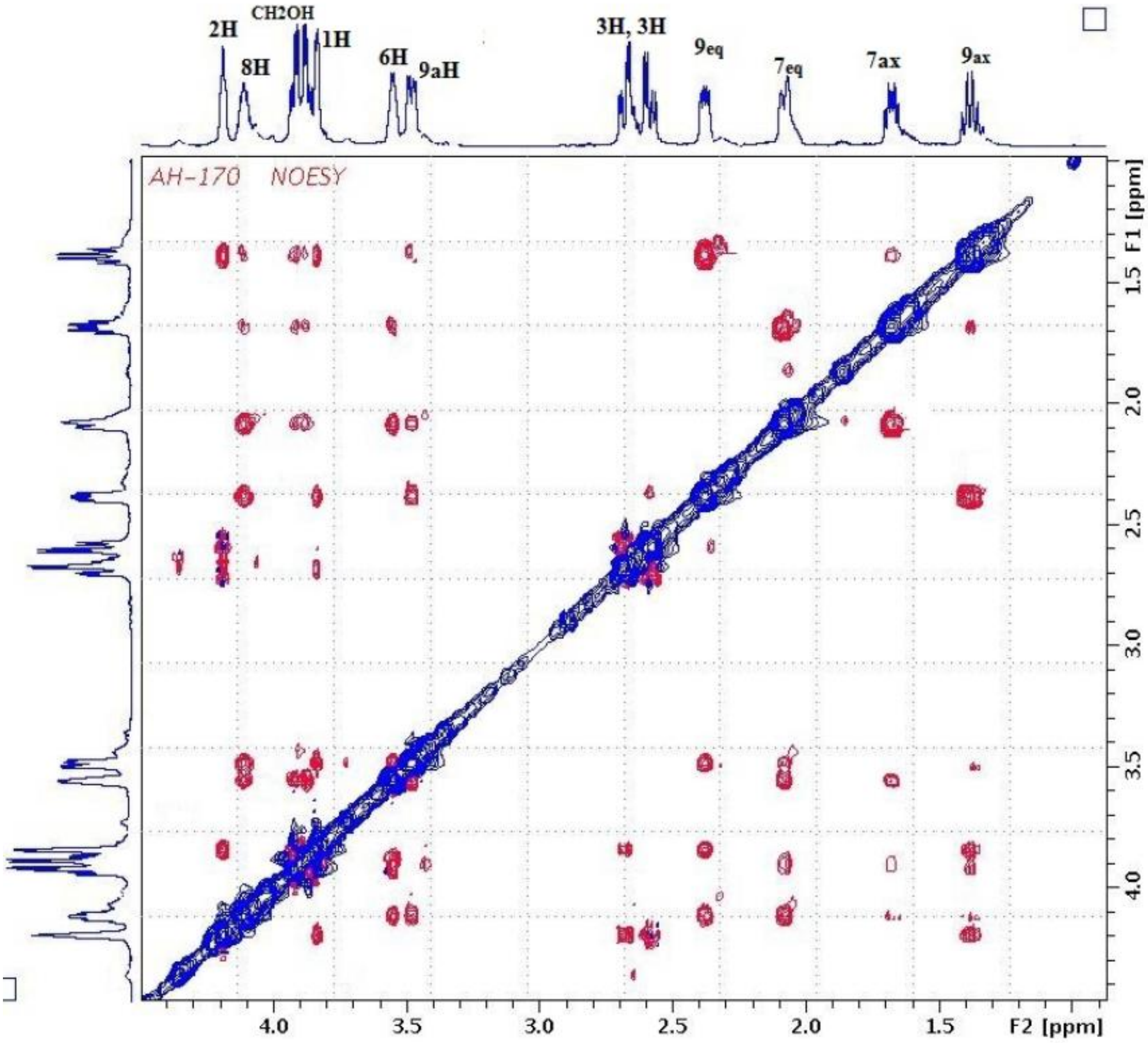

nOe correlations

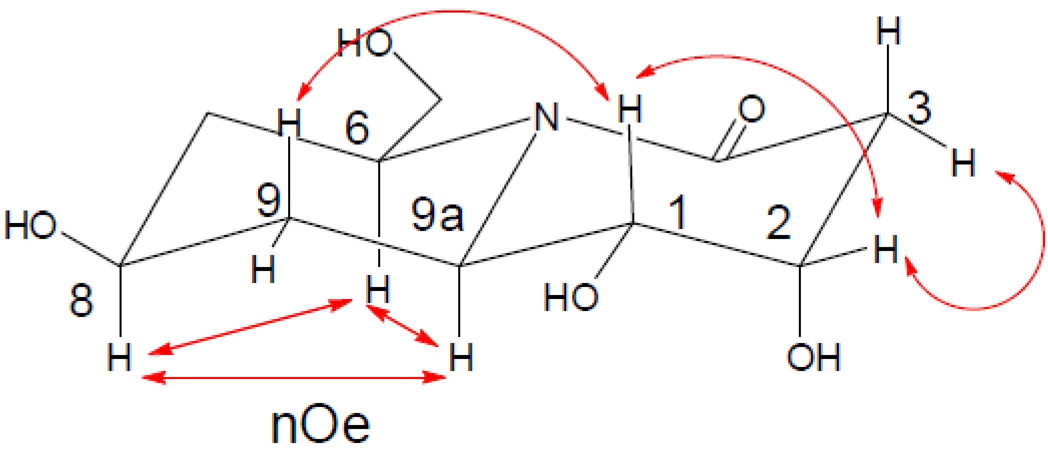

23
